# Loss of Pde1 function acts as an evolutionary gateway to penicillin resistance in *Streptococcus pneumoniae*

**DOI:** 10.1073/pnas.2308029120

**Published:** 2023-10-05

**Authors:** Carolin M. Kobras, William Monteith, Sophie Somerville, James M. Delaney, Imran Khan, Camilla Brimble, Rebecca M. Corrigan, Samuel K. Sheppard, Andrew K. Fenton

**Affiliations:** ^a^School for Biosciences, Florey Institute for Host-Pathogen Interactions, University of Sheffield, Sheffield S10 2TN, United Kingdom; ^b^Department of Biology, Ineos Oxford Institute for Antimicrobial Research, University of Oxford, Oxford OX1 3SZ, United Kingdom

**Keywords:** antimicrobial resistance, β-lactam antibiotics, *Streptococcus pneumoniae*, cyclic-di-AMP, population genomics

## Abstract

*Streptococcus pneumoniae* is a major human pathogen and rising resistance to β-lactam antibiotics, such as penicillin, is a significant threat to global public health. Mutations occurring in the penicillin-binding proteins (PBPs) can confer high-level penicillin resistance but other poorly understood genetic factors are also important. Here, we combined strictly controlled laboratory experiments and population analyses to identify a new penicillin resistance pathway that is independent of PBP modification. Initial laboratory selection experiments identified high-frequency *pde1* mutations conferring *S. pneumoniae* penicillin resistance. The importance of variation at the *pde1* locus was confirmed in natural and clinical populations in an analysis of >7,200 *S. pneumoniae* genomes. The *pde1* mutations identified by these approaches reduce the hydrolytic activity of the Pde1 enzyme in bacterial cells and thereby elevate levels of cyclic-di-adenosine monophosphate and penicillin resistance. Our results reveal rapid de novo loss of function mutations in *pde1* as an evolutionary gateway conferring low-level penicillin resistance. This relatively simple genomic change allows cells to persist in populations on an adaptive evolutionary pathway to acquire further genetic changes and high-level penicillin resistance.

## Significance

*Streptococcus pneumoniae* is an important bacterial pathogen responsible for many serious infections worldwide. Infections are often treated with penicillin antibiotics. This provides a selection pressure for the emergence of resistant strains over time, reducing treatment options and threatening patients. Combining lab evolutionary and comparative genomics approaches, we identify unique loss of function mutations in the Pde1 enzyme that lead to penicillin resistance in the absence of classical resistance determinants. We confirm this effect across clinical isolates and characterize the impact of natural genetic variation on Pde1 function. Characterization of end-stage penicillin resistance genes has, so far, not led to effective mitigations. Here, by characterizing the evolutionary events leading toward resistance, we open different possibilities for interventions against resistant *S. pneumoniae.*

*Streptococcus pneumoniae* is a major human pathogen and causes over 1 million deaths each year (1, 2). *S. pneumoniae* infections are commonly treated with β-lactam antibiotics, such as penicillin, amoxicillin, or ampicillin, but an alarmingly high number of strains have developed resistance to this global front-line treatment strategy (3–5). High levels of penicillin resistance in *S. pneumoniae* are classically associated with dramatic changes to the drug target, which are enzymes named penicillin-binding proteins (PBPs) that have important functions in cell wall synthesis (6, 7). Resistance involves alterations to the underlying *pbp* genes and the introduction of large sequence blocks of divergent genetic material originating from other *Streptococcus* species (7, 8). The resulting chimeric, or mosaic, PBP proteins retain their essential cell wall synthetic functions but have lower binding affinity for β-lactams. In this way, the acquisition of mosaic-PBPs has been the paradigm for gaining resistance against penicillin antibiotics in *S. pneumoniae* since their discovery in the 1970s (6).

Whilst studies of *S. pneumoniae* penicillin resistance have focused on mosaic-PBPs, the acquisition of these genes is not always sufficient for high levels of resistance, with other genes also identified as important (9–11). For example, the two-component regulatory system CiaRH (12, 13) and mutations in the *murMN* operon are linked to penicillin resistance (14, 15). As more genomic data becomes available, understanding of the genetics underlying penicillin resistance has dramatically increased (11, 16), with comparative genomic analyses identifying numerous associated genetic variations (7, 17). However, many questions remain about covariation and interactions among associated genetic elements, their clinical significance, and the order of their acquisition. Here, we combined laboratory evolution experiments with comparative genomics of natural populations to find that novel mutations in the gene called *pde1* lead to penicillin resistance in *S. pneumoniae*. We propose *pde1* be considered a new penicillin resistance determinant in *S. pneumoniae* and characterize the effect natural variation in this gene has on the cell.

**Fig. 1.**
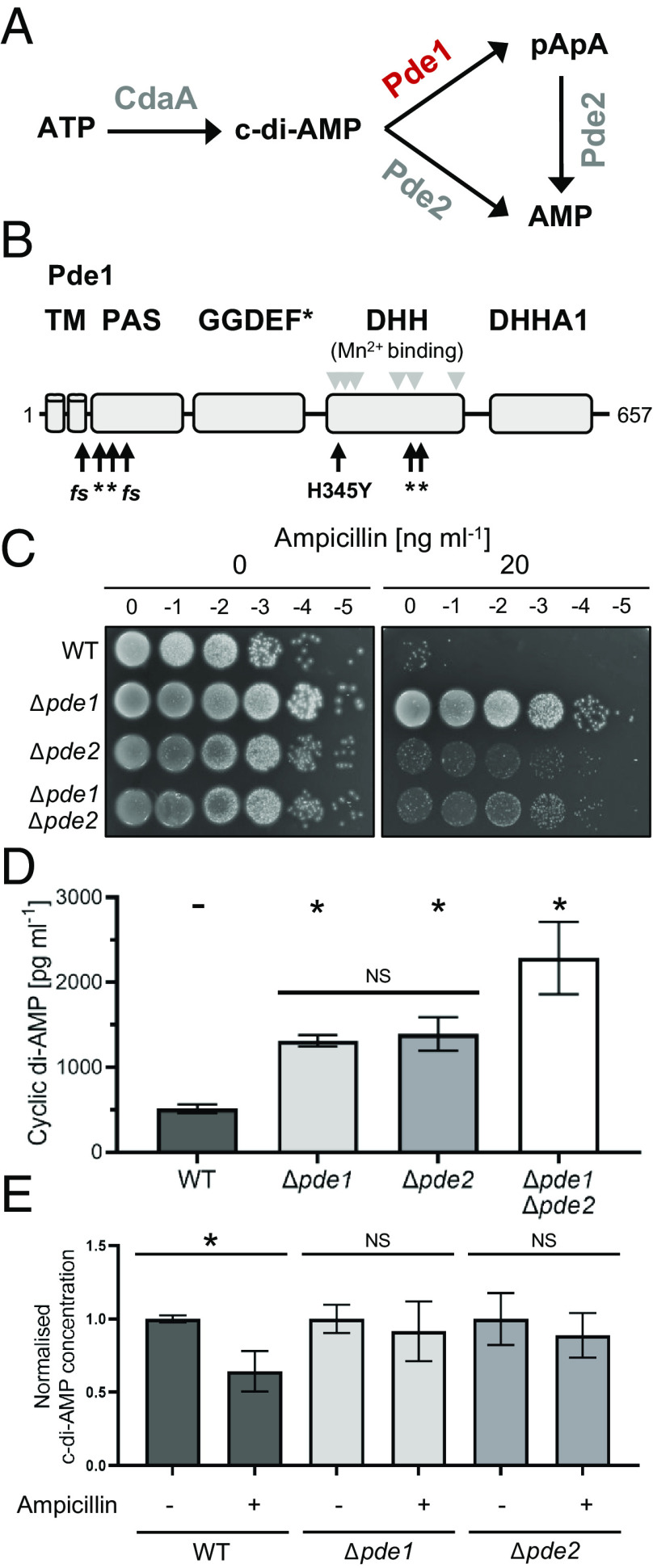
Loss of Pde1 or Pde2 increases resistance to ampicillin. (*A*) Overview of the c-di-AMP synthesis pathway in *S. pneumoniae.* The cyclase CdaA (DacA, SPD_1392) synthesizes the second messenger c-di-AMP from ATP. Pde1 (SPD_2032) linearises c-di-AMP to phosphoadenylyl adenosine (pApA). Pde2 (SPD_1153) cleaves pApA to two molecules of AMP but can also use c-di-AMP as substrate. Adapted from ref. 19. (*B*) The *pde1* mutations identified in *S. pneumoniae* strains selected for low-level ampicillin resistance cluster in the functional domains of Pde1. Domain prediction of Pde1 with mutations identified in this study indicated by black arrows. *fs:* frame-shift mutation. *: premature stop codon. The H345Y substitution is the result of a C1035T mutation. Pde1 protein domains are labeled: TM: Predicted transmembrane helix, PAS: Per-Arnt-Sim domain, GGDEF*: atypical GGDEF domain. DHH: DHH phosphatase family domain, and DHHA1 = DHH associated domain. Residues predicted to be important for Mn^2+^ co-factor binding are indicated by gray triangles. Information adapted from ref. 19 with additional information from Uniprot: A0A0H2ZQZ4_STRP2. (*C*) Deletion of *pde1* or *pde2* increases ampicillin resistance in a spot dilution assay. Strains were grown to mid-exponential phase, diluted to OD_600_ = 0.2 (0) and further serially diluted to 10^−5^ (−5). 5 µL of each dilution was spotted onto TSA plates containing 5% horse blood and the antibiotic concentration indicated in the figure. Plates were incubated for 20 to 24 h at 37°C in 5% CO_2_. The displayed plates are representative for n ≥ 6 independent repeats each showing the same effect. Note, a *pde1 pde2* double mutant shows a similar resistance phenotype and growth defect to the *pde2* single mutant (*SI Appendix*, Figs. S2 and S3). Additional polar effect controls are shown in *SI Appendix*, Fig. S6. (*D*) Deletion of *pde1* or *pde2* increases the c-di-AMP levels in the cell. Strains were grown to mid-exponential phase, harvested at OD_600_ = 0.5, and lysed. Intracellular c-di-AMP concentrations were quantified using an ELISA and normalized to the total protein concentration of the whole-cell lysates. Deletion of *pde1* or *pde2* increased the c-di-AMP concentration by over two-fold compared to WT levels. Highest c-di-AMP levels (~3.5-fold increase) were observed in a *pde1 pde2* double mutant. n = 3. (*E*) Exposing *S. pneumoniae* to sub-MIC concentration of ampicillin lowers cyclic-di-AMP concentrations in WT but not significantly alter in Δ*pde1* and Δ*pde2* strains. Strains grown in the presence of 15 ng mL^−1^ ampicillin as this represents ½ MIC for WT in liquid culture. Intracellular c-di-AMP concentrations were quantified using an ELISA and normalized to the untreated condition for each strain. Raw data and sampling information is given in *SI Appendix*, Fig. S4. n = 3. *P > 0.05, NS = no significant change.

Pde1 is involved in bacterial second messenger signaling by reducing the cyclic di-AMP (di-adenosine monophosphate, c-di-AMP) concentration in the cell. Since its discovery over a decade ago (18), changes in cellular c-di-AMP concentrations have been linked to a variety of nonantibiotic-related phenotypes and stress responses in *S. pneumoniae* (19–22). The c-di-AMP molecule is generated from two Adenosine Tri Phosphate (ATP) molecules by the cyclase CdaA (DacA, SPD_1392, see Fig. 1*A*). To reduce the c-di-AMP concentration in the cell, the Pde1 phosphodiesterase enzyme (SPD_2032) cleaves c-di-AMP into the linear molecule phosphoadenylyl adenosine (5′-pApA) (19). This protein is an ortholog of GdpP found in *Staphylococcus aureus* (18) or other Bacillota (formally Firmicutes) bacteria like *Bacillus subtilis* (23) and *Enterococcus faecalis* (24, 25). The Pde1 protein contains two N-terminal transmembrane helices, a PAS (Per-Arnt-Sim) sensory domain, and a degenerated GGDEF domain (Fig. 1*B*). Yet, for its in vitro c-di-AMP hydrolytic function, only the DHH and DHHA1 domains are required (19).

Reduction of Pde1 function in *S. pneumoniae* has been previously linked to survival in various conditions. These include a higher susceptibility to DNA damage, defective ion homeostasis, reduction in competence, as well as to lower tolerance to heat, acidic, and osmotic stress (19–21). Pde1 is also required for full virulence in mouse and rabbit infection models (19, 26–28). In addition to Pde1, the *S. pneumoniae* genome encodes a second phosphodiesterase termed Pde2 (SPD_1153, also called PapP, 29). Similarly to Pde1, Pde2 hydrolyzes c-di-AMP but also uses the linear molecule 5′-pApA as substrate, in each case forming AMP and thereby returning the signaling molecule back to the metabolic nucleotide pool (Fig. 1*A*, 19). More recently, cells lacking Pde2, but not Pde1, stimulated induction of interferon β and led to hyperactivation of the macrophage responses during infection. This suggests that Pde1 and Pde2 have distinct functions in the cell despite sharing similar c-di-AMP phosphodiesterase activities (19, 30).

In this study, we identified an association between the second messenger c-di-AMP and increased levels of penicillin resistance against cell wall targeting antibiotics in *S. pneumoniae.* Laboratory evolution experiments and genome sequencing revealed that loss of function mutations in the *pde1* gene conferred low-level ampicillin resistance. To study the clinical significance of these data, we extended this observation to natural populations and analyzed >7,200 *S. pneumoniae* genomes. This identified five common mutations in *pde1* associated with penicillin resistance across these populations. To gain a mechanistic understanding of how loss of function changes in Pde1 lead to penicillin resistance, we tested each of the substitution variants identified and revealed that they all reduce Pde1 hydrolytic activity, thereby increasing the concentration of c-di-AMP in the cell. Our results suggest loss of Pde1 function may act as an evolutionary gateway toward increased penicillin resistance in *S. pneumoniae*, allowing cells to survive at low antibiotic concentrations to later thrive in these conditions after acquiring additional genetic changes and complementary resistance mechanisms.

## Results

### Loss of Pde1 c-di-AMP Phosphodiesterase Function Increases Ampicillin Resistance.

To identify novel genetic loci that support penicillin resistance, we took a laboratory evolution approach. For this, we exposed *S. pneumoniae* cells to ampicillin, a member of the penicillin group of antibiotics. We generated ampicillin slope plates, selecting for spontaneous resistance mutants along a gradient that ranged from 0 to a maximum of 62.5 ng mL^−1^ ampicillin. The maximum concentration of 62.5 ng mL^−1^ was chosen as it represents the “susceptible” to “intermediate” penicillin resistance boundary by international clinical standards (*SI Appendix*, Fig. S1*A*, 31).

This laboratory evolution approach generated 20 independent mutants. All strains showed increased ampicillin resistance and no mutations in any *pbp* genes or previously known resistance determinants (*SI Appendix*, Fig. S1*B*). A comparative genomics analysis of these strains revealed that 40% of isolates contained mutations in the same gene (*SI Appendix*, Fig. S1*B*). This gene encodes the c-di-AMP phosphodiesterase *pde1* (*spd_2032*). The identified mutations were almost exclusively *pde1* nonsense or frameshift mutations (6 in total), with only one nonsynonymous mutation resulting in a H345Y substitution (*SI Appendix*, Fig. S1*B*). This substitution was located in the DHH domain, which is the region of Pde1 responsible for c-di-AMP hydrolysis and is predicted to be important for Mn^2+^ cofactor binding (23). This suggested that the H345Y substitution also results in either the reduction or loss of Pde1 hydrolytic activity. Combined, these findings suggest that loss of Pde1 function is responsible for the increased ampicillin resistance phenotype. To confirm this observation, we generated a *pde1* deletion mutant and tested its resistance to ampicillin using a viability assay. Consistent with our expectation, deletion of *pde1* increased the ampicillin resistance in our viability assays (Fig. 1*C*).

The Pde1 enzyme is known to degrade the c-di-AMP second messenger in *S. pneumoniae*, hydrolyzing it to 5′-pApA before a secondary hydrolysis step cleaves this intermediate into two AMP molecules (Fig. 1*A*, 19). Therefore, reducing Pde1 function increases c-di-AMP concentrations by lowering the hydrolysis rate in the cell (19). We confirmed this for our Δ*pde1* strain using an ELISA assay to measure the cytosolic c-di-AMP concentration. This assay showed that deletion of *pde1* increases c-di-AMP concentrations by approximately twofold (Fig. 1*D*). *S. pneumoniae* cells are typically surrounded by a polysaccharide capsule and this is often removed in lab-based molecular studies. Although uncommon, this can have an impact on cell growth in certain conditions. Indeed, we found deleting *pde1* reduced cell size by ≈15% in our wild-type (WT) strain without the capsule (*SI Appendix*, Fig. S2), although this phenotype was lost when the Δ*pde1* mutation was introduced to the encapsulated strain background (*SI Appendix*, Fig. S3). We also tested the Δ*pde1* mutant growth rate and found that it was not significantly altered, consistent with previously published observations (*SI Appendix*, Fig. S2) (26). We conclude that deletion of *pde1* increases ampicillin resistance and has no effect on growth rate or cell morphology when the polysaccharide capsule is present.

### Loss of Pde2 c-di-AMP Phosphodiesterase Function Increases Penicillin Resistance but Also Causes a Reduction in Cell Size.

The *S. pneumoniae* genome encodes a second enzyme capable of hydrolyzing c-di-AMP, Pde2, and previous studies have shown that loss-of-function of either enzyme increases the c-di-AMP pool in the cell to a similar degree (Fig. 1*D*) (19). Because of this, we reasoned that *pde2* loss of function might also increase resistance to the penicillin class of antibiotics. As predicted, a Δ*pde2* mutant led to an increase in ampicillin resistance (Fig. 1*C*). Next, we tested a Δ*pde1* Δ*pde2* double mutant, which gave a similar level of ampicillin resistance as Δ*pde1* but had fourfold higher levels of c-di-AMP compared to WT (Fig. 1 *C* and *D*). In both cases, the increased ampicillin resistance came at the cost of reducing both growth rate and cell size (*SI Appendix*, Fig. S2). To quantify the morphological changes, we measured the morphology of >2,000 Δ*pde2* and the Δ*pde1* Δ*pde2* double mutant cells. These were both significantly smaller than WT controls (*SI Appendix*, Figs. S2 and S3), showing a 30 to 40% reduction in cell size and a 20% reduction in both length and width (*SI Appendix*, Fig. S2). This reduction in size was less pronounced in an encapsulated strain background (15 to 20%), but this still represents a significant deviation from WT (*SI Appendix*, Fig. S3). We conclude that loss of *pde2* also increases penicillin resistance but the *S. pneumoniae* cell pays a greater fitness cost, evidenced by the reduction in overall cell size and growth rate in strains lacking capsule (*SI Appendix*, Figs. S2, S3, and S5).

### Maintaining High Levels of c-di-AMP Is Required for *S. pneumoniae* Survival at Sub-MIC (Minimum Inhibitory Concentration) Ampicillin Concentrations.

To measure the effect ampicillin has on c-di-AMP levels in *S. pneumoniae* cells, we challenged WT, Δ*pde1*, and Δ*pde2* strains with ampicillin concentrations lower than the MIC. Using an ELISA assay to quantify the c-di-AMP pool. This revealed sub-MIC concentrations of ampicillin cause a significant reduction in c-di-AMP concentration in WT cells (Fig. 1*E*). This reduction was mitigated by the removal of either phosphodiesterase enzyme Pde1 or Pde2 (Fig. 1*E*). This suggests the consequence of removing Pde1 or Pde2 from the cell is both an increase in the base c-di-AMP concentration and also a dampening of the c-di-AMP reduction effect when the cells are challenged with sub-lethal ampicillin concentrations.

### Loss of *pde1* and *pde2* Function Increases Resistance to Cell Wall–Targeting Antibiotics.

To examine the more widespread clinical significance of our ampicillin resistance measurements, we confirmed that our Δ*pde1* and Δ*pde2* strains were also more resistant to penicillin G (*SI Appendix*, Fig. S5). Penicillin G is used for standardized MIC testing (31) and is often used to treat *S. pneumoniae* infections in the clinic (3, 4). We found Δ*pde1* increased the penicillin G MIC by approximately twofold (from 6 to 12 ng mL^−1^), while Δ*pde2* increased the MIC to a slightly lower concentration of 8 ng mL^−1^ (*SI Appendix*, Fig. S5*C*). To test whether these increases were specific to the penicillin class of antibiotics or more broadly applicable to cell wall-targeting antibiotics, we repeated our MIC testing using vancomycin, a potent inhibitor of cell wall synthesis (*SI Appendix*, Fig. S5). We also tested both erythromycin and chloramphenicol, which act as inhibitors of protein synthesis (*SI Appendix*, Fig. S5). In these assays, we observed an increase in vancomycin resistance but no change in erythromycin or chloramphenicol resistance levels (*SI Appendix*, Fig. S5*C*). We conclude that increases in antibiotic resistance in the absence of the Pde1 and Pde2 function are specific to cell wall–targeting antibiotics.

In the *S. pneumoniae* genome, the *pde1* gene is found in an operon with *rplI* (*spd_2031*) with a short overlap between the two open reading frames, while *pde2* lies upstream of *rpmE2* [spd_1154, (32)]. To confirm the ampicillin resistance phenotype of both Δ*pde1* and Δ*pde2* strains was specific to each gene and not a product of downstream genetic effects, we repeated our ampicillin resistance and cell growth assays using Δ*rplI* and Δ*rpmE2* strains. These assays revealed no difference in resistance, no change in growth rate, and no change in cell morphology compared to WT (*SI Appendix*, Fig. S6). As an additional control, we tested the impact each of the antibiotic resistance cassettes used for strain construction had on our strains and found that none of them influenced ampicillin resistance (*SI Appendix*, Fig. S6*D*). These results confirm the ampicillin resistance phenotype is specific to disruption of the *pde1* and *pde2* loci.

### Genetic Variation in *pde1* Is Associated with Increased Penicillin Resistance in Circulating *S. pneumoniae* Populations.

**Fig. 2.**
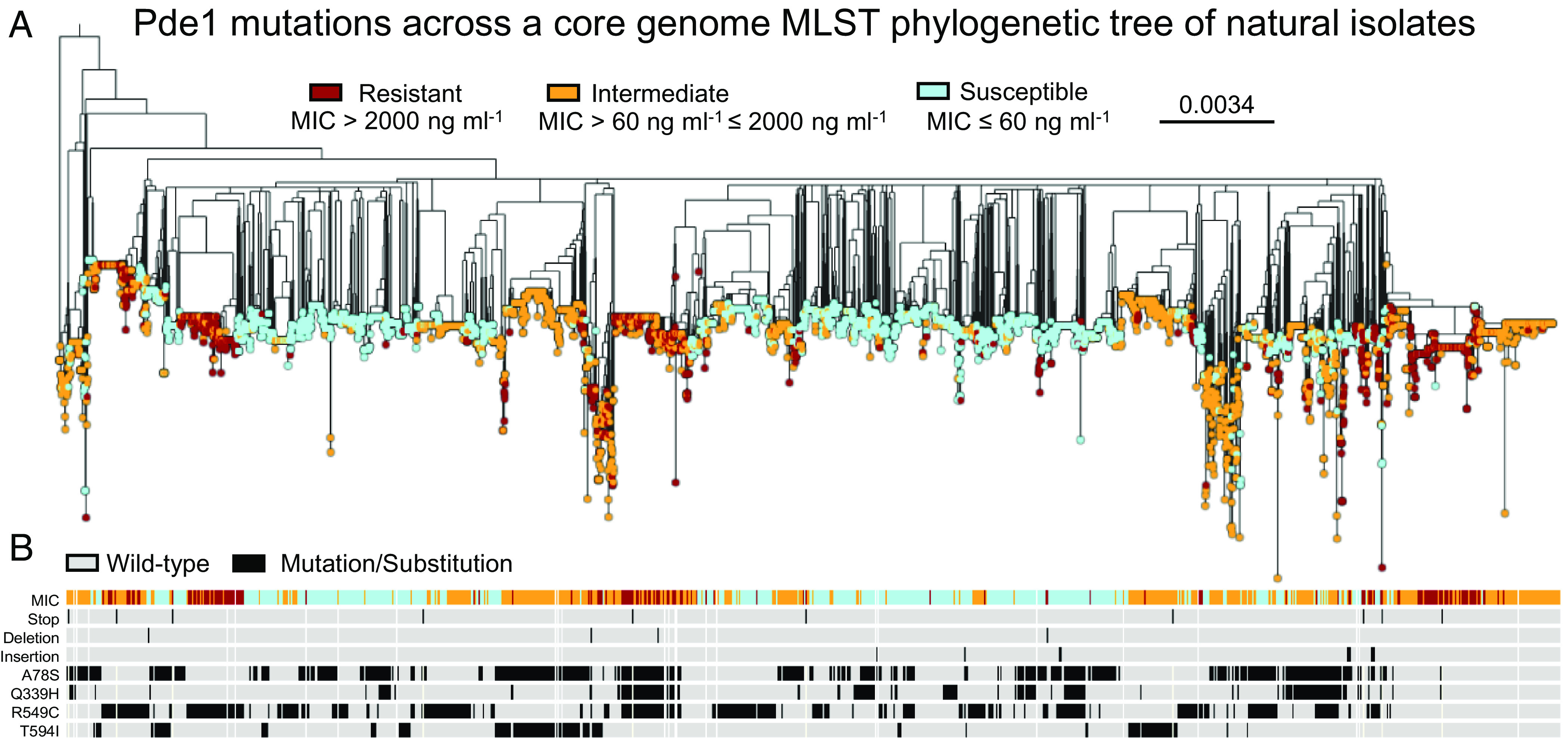
Sequence variation of Pde1 across *S. pneumoniae* natural populations. (*A*) MLST phylogenetic tree of >7,200 genome sequences of *S. pneumoniae* isolates retrieved from the PubMLST data base (33) and displayed using iTOL (35). Isolates were split into groups based on their penicillin resistance profile and using the EUCAST clinical breakpoints (31). The *pde1* mutations and substitution mutations found in this dataset are shown in their corresponding position below the tree, highlighted in black if present and gray if absent. (*B*) Strains containing premature stop codons, deletions, insertions, and the four most common substitution mutations: A78S, Q339H, R549C, and T594I are indicated (see Fig. 3 for frequency data). Pde1 sequence variation is not limited to specific lineages, mutations seem to be spread across the phylogenetic tree suggesting that they have occurred several times independently or have been transferred via horizontal gene transfer. Scale bar, average number of mutations per site.

**Fig. 3.**
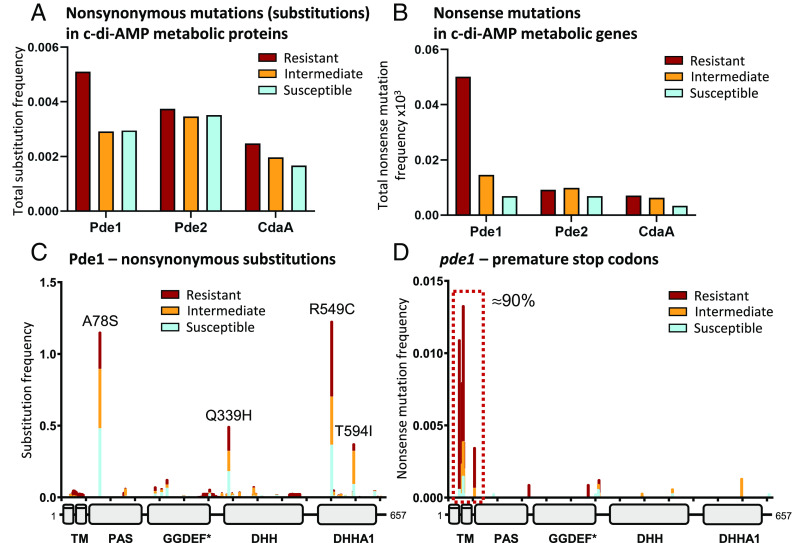
Variation of Pde1 sequences is common in penicillin-resistant isolates and clusters into five distinct groups. Phylogenetic analysis reveals the Pde1 protein has higher sequence of variation compared to other proteins involved in c-di-AMP metabolism (*A* and *B*). This increased variation clusters into five groups, four substitution mutations (*C*), and a cluster of nonsense mutations found early in the sequence (*D*). (*A*) Nonsynonymous substitution mutation frequency in c-di-AMP metabolic proteins within the phylogenetic dataset. Pde1-resistant isolates display the highest levels of sequence variation compared to other proteins in the same pathway. (*B*) Nonsense mutations are more frequent in Pde1-resistant strains. This increase was over fivefold higher than those found in Pde2- and CdaA-resistant strains. (*C*) Sequence variation for Pde1 at each amino acid position reveals four common nonsynonymous substitutions. Combined, these four mutations account for ≈ 52% of all Pde1 mutations found in the dataset. Amino acid changes are indicated where the mutation frequency is >0.25. (*D*) 90% of nonsense mutations found in *pde1* cluster within the region encoding the N-terminal transmembrane helixes (red box). Genome sequences and penicillin MIC categories are identical to those shown in Fig. 2. For *A* and *B*, all mutations were normalized by the number of protein sequences available for each group and adjusted for protein length. Pde1 protein domains are labeled as in Fig. 1*A*.

To broaden the significance of our laboratory evolution experiments, we aimed to understand the role *pde1* mutations play in penicillin resistance in circulating human carriage and clinical *S. pneumoniae* populations. We hypothesized that genes involved in c-di-AMP metabolism (*cdaA*, *pde1*, and *pde2*—Fig. 1*A*) would contain significant genetic variation associated with penicillin resistance in these isolates. A total of >7,200 *S. pneumoniae* genomes, available through the PubMLST database (33), were split into susceptible (MIC ≤ 62.5 ng mL^−1^), intermediate (62.5 ng mL^−1^ < MIC ≤ 2,000 ng mL^−1^), and resistant (MIC > 2,000 ng mL^−1^) groups, according to their reported penicillin MICs (Fig. 2). The amino acid sequences of Pde1, Pde2, and CdaA were analyzed for sequence variation in this population, normalized by the number of isolates in each resistance group, and adjusted for protein length. For nonsynonymous substitution mutations, as well as insertions and deletions (indels), we found that Pde1 and Pde2 had similar levels of sequence variation, and both were more variable than the c-di-AMP cyclase CdaA, which is consistent with reports that *cdaA* is essential (Fig. 3, 19, 34). Furthermore, mutations found in *pde1* were enriched in resistant strains, suggesting a link between sequence variation at this locus and penicillin resistance (Fig. 3*A*). However, more strikingly, we found a fourfold higher accumulation of nonsense mutations in *pde1* within the penicillin-resistant group (Fig. 3*B*). Of these, ≈90% of the mutations clustered very early in the *pde1* reading frame, predicted to truncate the protein within its N-terminal trans-membrane domains (Fig. 3*D*). As these mutations result in a premature stop of translation, Pde1 function would be abolished in these cells. In contrast, nonsense mutations were rarely detected in Pde2 or CdaA (Fig. 3*B* and *SI Appendix*, Fig. S7), consistent with the high fitness cost in cell viability, growth, and cell division these mutations would likely bring (19–21, 26–28).

Further analysis of amino acid sequence variation across Pde1, Pde2, and CdaA identified specific “hot spots”, where variation was common (Fig. 3*C* and *SI Appendix*, Fig. S7 *C* and *E*). For Pde1, four nonsynonymous substitutions were identified: A78S, Q339H, R549C, and T594I (Fig. 3*C*). To control for clonal inheritance of *pde1* mutations, nonsynonymous mutations, indels and nonsense mutations were plotted on a core-genome multilocus sequence typing (MLST) phylogenetic tree (Fig. 2*B*). While mutations were often shared by closely related strains, suggesting vertical clonal inheritance, each mutation is widely distributed horizontally across the population and shared between distantly related strains (Fig. 2*B*). This suggests that sequence variation detected in Pde1 has either evolved many times independently (homoplasy) or has been transferred between isolates by horizontal gene transfer.

To demonstrate the independent emergence of the four common Pde1 substitutions across the phylogeny, we reconstructed the ancestral character states for each of the four hot spot sites (*SI Appendix*, Fig. S8). To do this we used the most parsimonious reconstruction analysis which attempts to predict the minimum numbers of changes within a phylogeny required to describe the patterns observed at the tips. For this analysis, each node was treated as if it were the root of the phylogeny and the ancestral state of the four substitution sites was reconstructed generating a parsimony score. This score represents the minimum number of changes required to describe the distribution of each Pde1 variant data across the phylogeny. For the Pde1 variants A78S, Q339H, R549C, and T594I, these are 4145, 4999, 4386, and 5351, respectively (*SI Appendix*, Fig. S8). These figures suggest frequent and independent emergence of the *pde1* nonsynonymous mutations (de novo or through homologous gene transfer) in closely related bacteria must have occurred to explain these inheritance patterns. Taken together, our phylogenetic analyses show notable genetic variation exists across all c-di-AMP metabolic genes, with an overrepresentation of four Pde1 substitutions and a cluster of *pde1* nonsense mutations found in isolates resistant to penicillin. This suggests a link between *pde1* mutations and penicillin resistance in natural *S. pneumoniae* populations, isolated from carriage and infections from across the world.

### Loss of Pde1 Function Increases Penicillin Resistance in Clinical Isolates with Divergent Genetic Backgrounds.

**Fig. 4.**
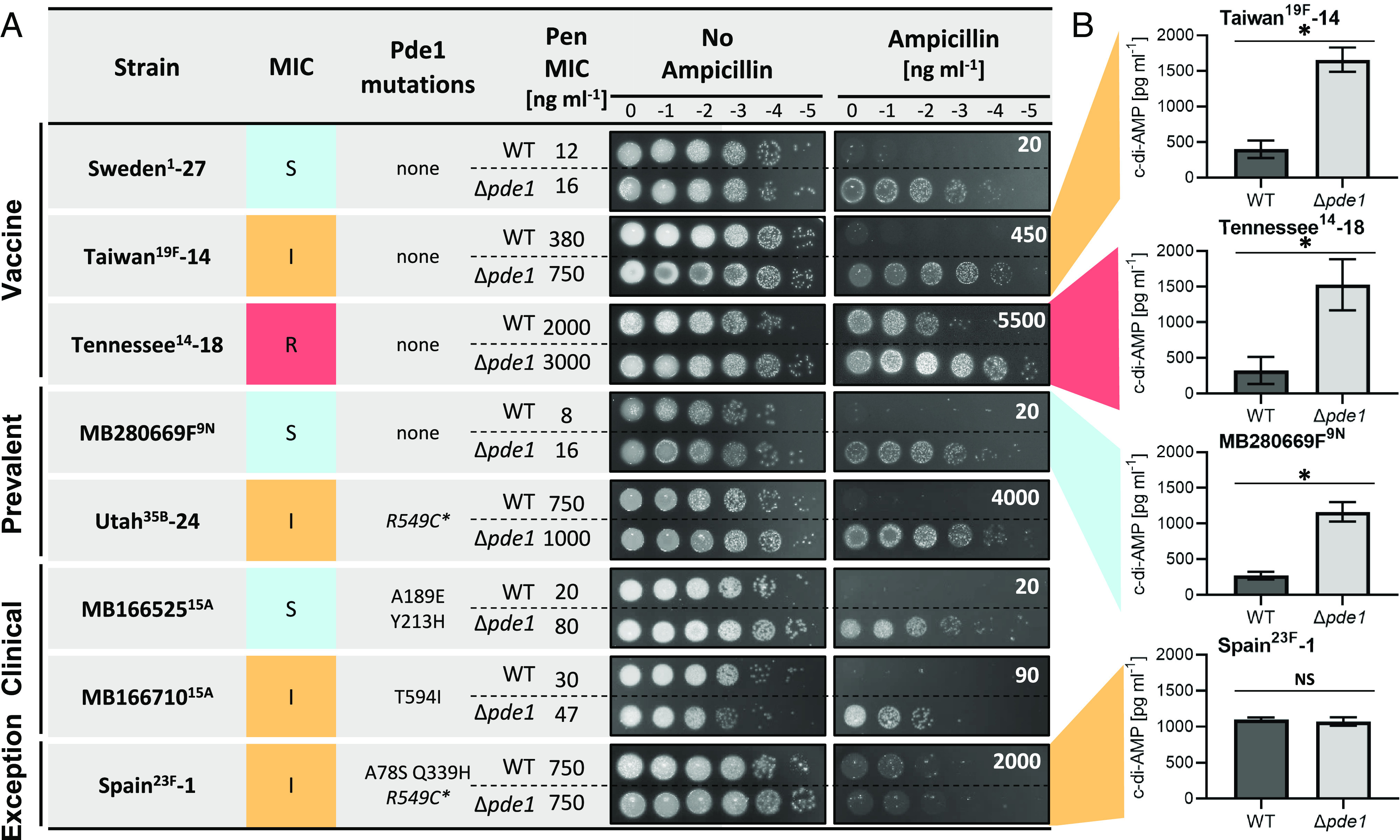
Loss of Pde1 function increases penicillin resistance in a range of clinical isolates. (*A*) Deletion of *pde1* led to increased penicillin and ampicillin resistance in clinical strains including serotypes covered by the PCV-13 vaccine, serotypes currently prevalent in the world (post-PCV13), and local clinical isolates. Importantly, this effect was observed in *S. pneumoniae* isolates classified as clinically sensitive, intermediate, or resistant to penicillin (31). Penicillin G MICs were measured using an Etest. Strain Spain^23F^-1 was included as an exception for which *pde1* deletion does not alter resistance. Serotypes for each strain are given in superscript. Where the strain is part of the Pneumococcal Molecular Epidemiology Network (PMEN, 39), its collection number is indicated by a dash following the strain name. An extended dataset and further strain information can be found in *SI Appendix*, Fig. S9. (*B*) c-di-AMP levels increase in Pde1 deficient clinical isolates. The c-di-AMP concentration of three clinical isolates, representative for the susceptible, intermediate, and resistant category, was elevated in *pde1* deletion strains compared to the WT. Deletion of *pde1* in Spain^23F^-1 did not increase c-di-AMP levels in the cell. * denotes a statistical significant change (*P* > 0.05), NS = not significant, - = the normalized WT dataset. n = 3.

**Fig. 5.**
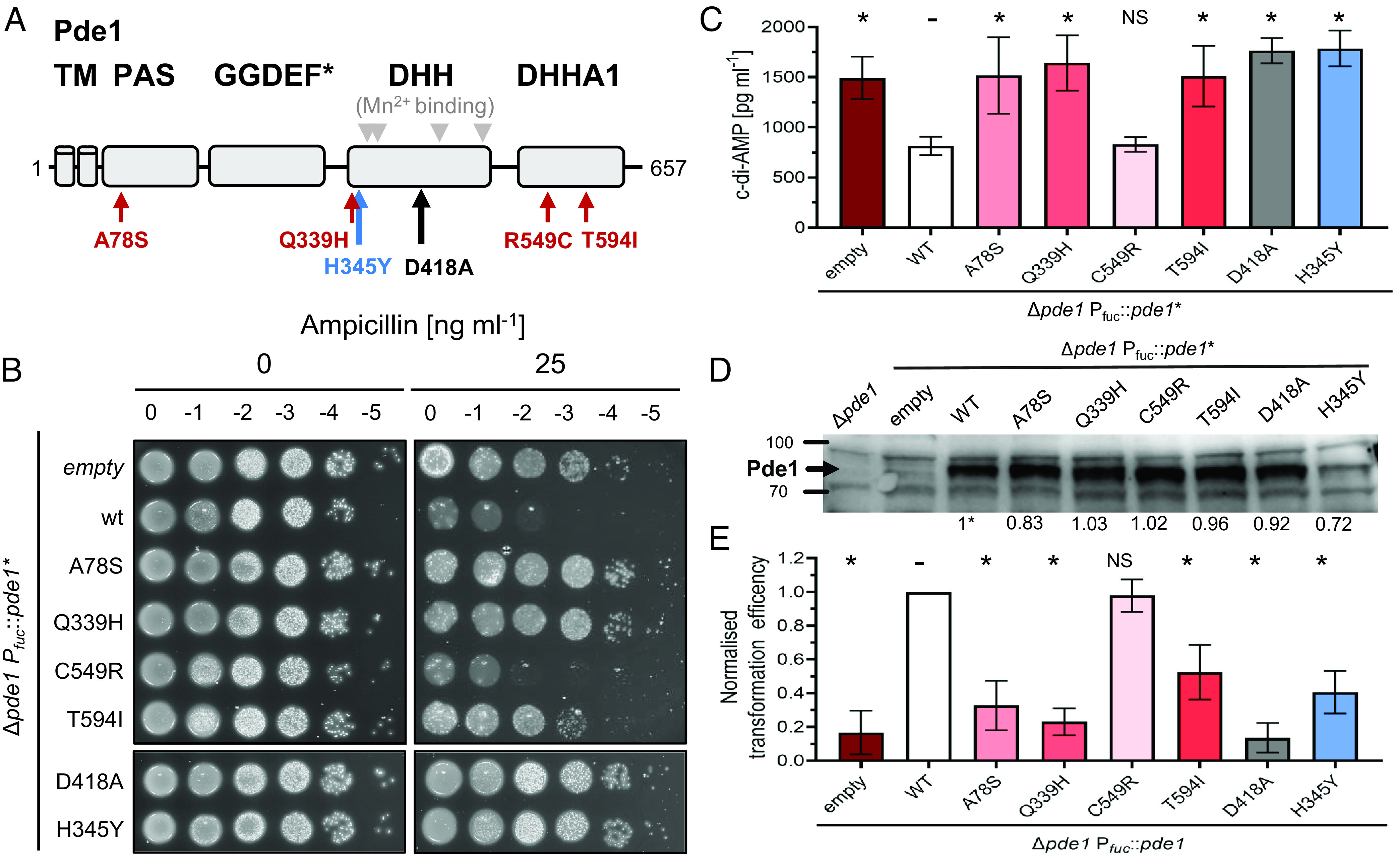
Functional characterization of Pde1 substitutions suggests penicillin resistance is gained through loss of c-di-AMP hydrolytic function. (*A*) Pde1 substitutions identified in *S. pneumoniae* clinical isolates cluster in functional domains. Domain prediction of Pde1 with “hot spot” substitutions identified in clinical samples indicated by red arrows. The Pde1 substitution identified in our laboratory evolution experiment is indicated in blue. A known loss of function mutation of Pde1 homologs is indicated in black (18, 23). Pde1 protein domains are labeled as in Fig. 1*A*. (*B*) Expression of Pde1 variants increases ampicillin resistance in a viability assay. Strains were grown to mid-exponential phase, diluted to OD_600_ = 0.2 (0) and further serially diluted to 10^−5^ (−5). Five µL of each dilution was spotted onto TSA plates containing 5% horse blood and the antibiotic concentration indicated in the figure. Plates were incubated for 20 to 24 h at 37 °C in 5% CO_2_. The displayed plates are representative of n ≥ 3 independent repeats, each showing the same effect. Expression of any Pde1 variant increases resistance, with the exception of the *pde1* C549R construct. (*C*) Elevated c-di-AMP concentrations in strains expressing Pde1 variants. The c-di-AMP concentration was measured using a ELISA assay on whole-cell lysates (n = 3). * denotes a statistical significant change compared to the WT complementation strain measured using a *t* test (*P* > 0.05), NS = not significant, - = the normalized WT dataset. (*D*) Anti-Pde1 immunoblot analysis of whole-cell lysates from the indicated strains. The position of protein markers are indicated in kiloDaltons, Pde1 ≈ 73 KDa. The numbers below each band represent normalized Pde1 protein levels. Representative blot shown, n = 4. (*E*) Transformation efficiencies of the indicated strains. Cells of each strain were treated to induce natural competence and transformed with 300 ng of gDNA containing a selectable marker. The number of transformations were enumerated and averaged. n ≥ 3. Note, for these experiments, our WT *pde1* allele contains the R549C mutation and this was reverted to C549R to test the effect this variant may have on Pde1 activity.

Our comparative genome analyses suggested that Pde1 loss of function mutations are enriched in circulating *S. pneumoniae* isolates. To validate this finding and confirm the clinical relevance of this dataset, we tested the effect Pde1 loss of function has on penicillin resistance in thirteen *S. pneumoniae* clinical isolates in a lab setting (*SI Appendix*, Fig. S9). To capture the widest possible diversity of *S. pneumoniae* isolates, we tested a variety of clinical serotypes. We chose some strains representing serotypes that are included in the pneumococcal PCV-13 conjugate vaccine (Fig. 4, 36). Whilst historically highly significant, the abundance of these serotypes has reduced in the population recently due to the introduction of the vaccine. Therefore, we also chose strains representing serotypes that represent some of the most common serotypes in current global circulation (37, 38). We additionally tested two further strains isolated from our local UK hospital. To capture a broad range of penicillin resistance characteristics, we selected strains representing penicillin-sensitive, intermediate, and resistant phenotypes, with, and without, evidence of PBP genetic mosaicism *SI Appendix*, Fig. S7 (mosaicism used as a classical indicator of penicillin resistance) (6). In all cases, the *pde1* gene was sequenced to identify any genetic variation at this site, and the effect Pde1 loss of function had on each strain was measured using penicillin G and ampicillin viability assays. The full dataset of all strains tested is presented in *SI Appendix*, Fig. S9, with representative examples shown in Fig. 4. In almost all cases, Pde1 loss of function increased ampicillin resistance in this diverse set of clinical strains (Fig. 4*A* and *SI Appendix*, Fig. S9). As a control, the growth rate of the Taiwan-14 and Spain-1 strains was measured to confirm that the Δ*pde1* mutations did not affect growth rate (*SI Appendix*, Fig. S10). Importantly, strains where the deletion of *pde1* had no effect on resistance already contained *pde1* alleles that we later found have reduced phosphodiesterase function (discussed later in this article, Fig. 5*C*). To confirm the effect our *pde1* deletion constructs had on c-di-AMP metabolism in these clinical isolates, we measured the c-di-AMP pools and found that, as expected, *pde1* deletion increased c-di-AMP concentrations in all cases where penicillin resistance was increased (Fig. 4*B*). We therefore conclude that regardless of the genetic background, serotype, or PBP mosaicism, Pde1 loss of function increase resistance to penicillin antibiotics.

### Circulating Nonsynonymous *pde1* Mutations Lead to Penicillin Resistance in *S. pneumoniae* Clinical Isolates.

To investigate the contribution each nonsynonymous *pde1* mutations had to increase resistance to penicillin antibiotics and gain a mechanistic understanding of Pde1 function, we tested the effect that each of the common substitutions (A78S, Q339H, R549C, and T594I) had on Pde1 activity in the *S. pneumoniae* cell. In addition, we also studied the H345Y variant identified in our lab evolution experiments (*SI Appendix*, Fig. S1*B*). As a control, we included the Pde1 active site mutation D418A, as previous work has shown this variant cannot hydrolyze c-di-AMP (18, 23). The D39 laboratory strain already carries the common R549C mutation, so we decided to revert this mutation to C549R to test the potential effect this variant might have on Pde1 activity. The locations of each of these protein variants on the Pde1 primary structure are summarized in Fig. 5*A*.

First, and importantly, we verified the genetic relationship between ampicillin sensitivity and *pde1* by constructing a strain deleted for *pde1* and harboring a fucose-inducible copy of *pde1* placed at an ectopic locus. As expected, fucose-induced expression of *pde1* reduced ampicillin resistance in our viability assays suggesting that *pde1* loss-of-function is the cause of the observed resistance phenotype under these conditions (Fig. 5*B*). Second, we tested the effect each Pde1 substitution had on ampicillin resistance. In almost all cases, ectopic expression of these modified *pde1* alleles failed to complement the ampicillin resistance phenotype (Fig. 5*B*). The only exception was the C549R variant that showed strong complementation and therefore nearly full restoration of ampicillin sensitivity (Fig. 5*B*). In this specific case, we conclude the C549R variant is likely a phenotypically neutral allele under the conditions tested (*Discussion*). Yet, it is our main conclusion that each Pde1 substitution identified in this study (A78S, Q339H, H345Y, and T594I) is capable of increasing ampicillin resistance validating our original genetic screen and our phylogenetic approach.

### Circulating Pde1 Substitutions Reduce Pde1 Cyclic Nucleotide Hydrolase Activity.

As each Pde1 variant maintained ampicillin resistance in our viability assays (Fig. 5*B*), we reasoned that these substitutions might reduce the cyclic nucleotide hydrolase activity of Pde1. Therefore, the activity of each Pde1 variant was measured using a c-di-AMP ELISA (Fig. 5*C*). Strikingly, when the Pde1 A78S, Q339H, T549I, and H345Y variants were expressed, they all showed increased c-di-AMP concentrations similar to empty vector and known loss of function controls (Fig. 5*C*). This suggests that each variant significantly reduces Pde1 function in vivo (Fig. 5*C*, full dataset and statistics *SI Appendix*, Fig. S11). As expected from the viability data (Fig. 5*B*), complementation using the *pde1* C549R variant showed no difference in c-di-AMP concentrations compared to *pde1*^wt^. This is consistent with the C549R variant being phenotypically neutral (Fig. 5*C*). To address the possibility that our observations were a result of differences in *pde1* expression, we carried out western blot analysis of each expression strain using anti-Pde1 antibodies (Fig. 5*D*) (19). These data confirm that expression of each *pde1* variant was similar to that of the *pde1*^wt^ control (Fig. 5*D*—quantification measures shown underneath each band). We conclude the Pde1 variants identified in this study reduce in vivo phosphodiesterase activity and increase ampicillin resistance.

### Circulating Pde1 Substitutions Reduce *S. pneumoniae* Natural Competence.

As loss of Pde1 activity results in ampicillin resistance, and these mutations are common in the *S. pneumoniae* phylogenetic tree, one may question why the *pde1* gene is maintained in *S. pneumoniae* populations at all. *S. pneumoniae* cells are naturally competent and can acquire DNA from the environment through horizontal gene transfer, which can introduce beneficial genetic variation (7). Based on previous observations (21), we hypothesized that our Pde1 loss of function variants might reduce transformation frequencies by inhibiting natural competence. To test this, we expressed each Pde1 variant in *S. pneumoniae* cells treated with a quorum sensing molecule (CSP-1) to induce competence and measured the transformation efficiency (Fig. 5*E*). In all cases, each Pde1 variant reduced the transformation efficiency compared to a WT control. As expected, the empty vector and D418A loss of function controls showed the lowest transformation efficiencies. However, each Pde1 variant exhibited a two- to three-fold reduction in the number of transformants recovered. Again, the only exception was the C549R variant, which had no significant effect on transformation efficiency. We conclude that transformation efficiency is reduced when Pde1 loss of function variants is expressed in the cell.

## Discussion

**Fig. 6.**
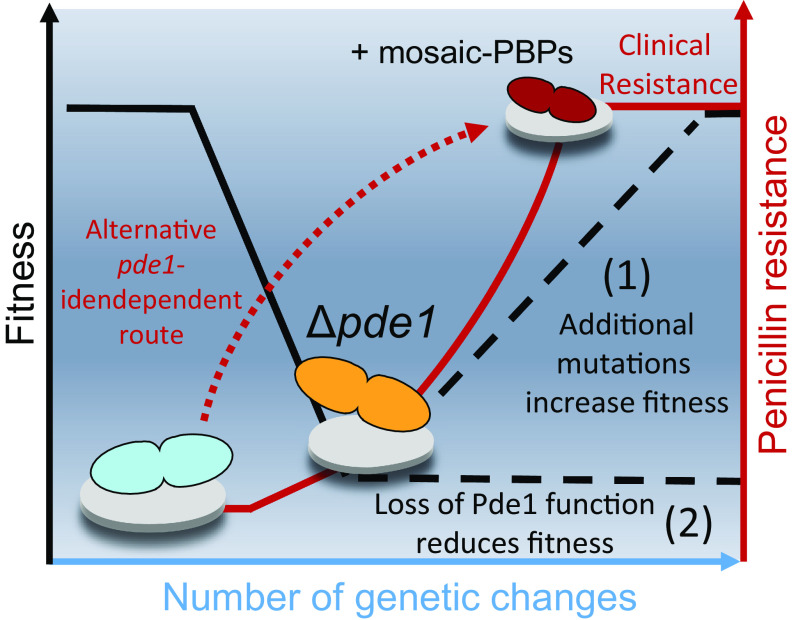
Loss of Pde1 function could serve as an evolutionary gateway toward penicillin resistance in *S. pneumoniae*. As penicillin antibiotic selection is applied to *S. pneumoniae* natural populations, loss of function mutations in *pde1* (and other genes) allow the cells to resist antibiotic challenges at relatively low antibiotic concentrations. This provides the opportunity for these cells to accumulate further genetic changes needed for clinical levels of resistance, which includes the acquisition of other mutations such as highly divergent “mosaic” penicillin-binding protein alleles (1). Alternatively, cells may be unable to overcome the fitness cost of Pde1 loss-of-function which leaves the cells vulnerable to extinction (2).

Evolutionary adaptation to resist antibiotic treatments is a continually emerging problem in *S. pneumoniae*. Current *S. pneumoniae* infection control involves large-scale vaccination efforts with vaccines targeting the polysaccharide capsule. This approach has successfully reduced the targeted *S. pneumoniae* capsule types thereby reducing antibiotic resistance in the short and medium term (40). However, after vaccine deployment, nontargeted capsule types will ultimately fill the vacated niche and start the process of acquiring antibiotic resistance over time (41, 42). Understanding this continual emergence of *S. pneumoniae* antibiotic resistance requires investigation of the possible evolutionary trajectories open to these strains. Here, we have identified Pde1 loss of function as a crucial evolutionary gateway toward penicillin resistance in *S. pneumoniae* and have quantified one of the fitness costs faced by cells that take this evolutionary route (Fig. 6).

Loss of function mutations in Pde1 occurred regularly in our laboratory experiments when *S. pneumoniae* cells were exposed to ampicillin. Cells lacking *pde1* were resistant to cell wall targeting antibiotics and showed a doubling of the penicillin MIC value (43). We went on to further characterize the effect Δ*pde1* had on growth, morphology, and the c-di-AMP concentration within the cell. Consistent with our laboratory screen, loss of Pde1 function increases resistance against the cell wall targeting antibiotics: ampicillin, penicillin, and vancomycin. Loss of Pde2 activity can also give rise to cell wall targeting antibiotic resistance but comes with a reduction in cell size and growth rate. Using quantitative measurement of c-di-AMP concentrations, we found the presence of ampicillin causes a decrease in c-di-AMP levels in WT cells. This effect is reduced by mutations that inactivate either of the two phosphodiesterase enzymes. Given the role of c-di-AMP in maintaining osmotic balance through potassium channels (20, 44), this reduction in c-di-AMP could contribute to bacterial death in the presence of cell wall-targeting antibiotics. Cells able to maintain high c-di-AMP levels have lower internal potassium concentrations and have less turgor pressure on their cell walls mitigating the effect of antibiotic-induced cell wall damage (20, 44–46). Therefore, having a higher starting c-di-AMP pool and lacking the enzymatic activities required to lower this pool might be the mechanism by which *pde1* and *pde2* cells survive in the presence of these antibiotics. Future work will focus on how c-di-AMP levels change in response to antibiotics across species and the impact our Pde1 variants have on potassium metabolism.

Building on our lab-based findings, we carried out a comparative genomics analysis of >7,200 *S. pneumoniae* isolates and showed that *pde1* mutations are associated with penicillin resistance in natural populations. While the distribution of nonsense mutations in the genomics analysis resembled those observed in the laboratory evolution experiment, the frequency of these mutations in natural populations was much lower. Instead, four “hot spot” nonsynonymous mutations were identified that emerged many times independently in *S. pneumoniae* populations. Three of these Pde1 substitutions (A78S, Q339H, and T594I) conferred reduced Pde1 phosphodiesterase function in vivo, significantly increasing c-di-AMP concentrations in the cells. The expression of each variant led to increased ampicillin resistance but also came at the cost of a reduction in competence.

To explain the Pde1 evolutionary tradeoff, we propose a model where the acquisition of *pde1* loss of function mutations is important for the initial stages of penicillin resistance, perhaps facilitating survival at low penicillin doses. However, strains lacking *pde1* are more sensitive to environmental stresses and so are less fit. This is supported by our cell morphology characterizations alongside other studies in the literature which show that *pde1* is required for survival in a number of hostile conditions (19, 20, 22). In addition, studies using animal models of infection have shown that *pde1* is required for virulence in both mouse pneumonia and rabbit meningitis models (19, 28, 37). Furthermore, the lower competence among cells expressing Pde1 variants and reduces the likelihood of horizontal gene transfer, an essential process for the acquisition of mosaic-PBPs and other antibiotic resistance determinants from the environment (11). The competence regulon is known to control the expression of essential virulence factors in *S. pneumoniae* (47) and therefore the reduction in competence measured here is likely indicative of *pde1* reduced in virulence. Future work will explore the impact our Pde1 variants have on gene expression. Taken together the evidence suggests Pde1 loss of function substitutions will have a detrimental impact on long-term fitness. Meaning *S. pneumoniae* lineages lacking Pde1 activity will be under selection pressure to reacquire a WT *pde1* allele from another donor via horizontal gene transfer or more likely acquire additional genetic changes to mitigate the *pde1* fitness cost (Fig. 6). In this model, Pde1 loss of function acts as an evolutionary gateway representing a costly genetic change in the long-term but allows a significant jump in penicillin resistance in the short-term when populations of cells are faced with an existential antibiotic challenge (Fig. 6).

Our model of Δ*pde1* acting as an evolutionary gateway suggests *pde1* mutations ultimately aid the acquisition of mosaic-PBPs by *S. pneumoniae*. In support of this model, there is some evidence for Δ*pde1* enabling the acquisition of mosaic-PBP alleles in the emerging literature. A recent CRISPRi-TnSeq-based study has shown *pde1* deletion reduces the fitness cost of low Pbp2x function (46). Taken to the population level, this would allow cells lacking Pde1 activity to acquire less functional mosaic-*pbp* alleles as they require less of PBP enzyme activity to remain viable. Applying this hypothesis to our phylogenetic data, we observe that strains containing our known *pde1* loss of function mutations have increased genetic diversity in their *pbp* alleles suggesting some of the evolutionary constraints on these genes has been removed (*SI Appendix*, Fig. S12). Our model also proposes *pde1* loss of function mutations precede mosaic-*pbp* acquisition and are not likely secondary compensatory events. To this point, our laboratory evolution data shows *pde1* mutations can occur spontaneously in *S. pneumoniae* to cause penicillin resistance independent of any *pbp* mutations (*SI Appendix*, Fig. S1 and Dataset S1). Also, we find *pde1* loss of function mutations in many clinical strains lacking mosaic-*pbp* alleles (*SI Appendix*, Fig. S9). However, we also know mosaic-*pbps* can be acquired without *pde1* mutations from our phylogenetic observations and from other studies (11). Whilst we do not exclude the possibility *pde1* mutations are compensatory secondary events after mosaic-pbp acquisition, we suggest Pde1 loss of function likely represents the first event in an evolutionary pathway to penicillin resistance.

In our complementation experiments, each of the Pde1 natural variants had similar effect on ampicillin resistance and transformation efficiency. However, and interestingly, the T594I variant showed a slightly more subtle phenotype change compared to the others, maintaining some c-di-AMP hydrolytic function and an intermediate reduction in transformation efficiency. This came at the cost of slightly less resistance in ampicillin viability assays (Fig. 5). The significance of the fitness trade-off differences between T594I vs A78S and Q339 are currently unclear. This is mainly due to our limited understanding of *S. pneumoniae* c-di-AMP signaling and how this affects the cell metabolism. In other bacteria, enzymes involved in c-di-AMP metabolism have dual functions. For example, GlmM acts as inhibitor of the c-di-AMP cyclase CdaA and is also an important enzyme in the cell wall precursor biosynthesis in other Bacillota (48, 49). For *S. pneumoniae*, further work is needed to explain how Pde1 loss of function alters wider cell metabolism and potentially cell envelope biology (45) to resist penicillin.

The finding that altered c-di-AMP metabolism is linked to penicillin resistance in *S. pneumoniae* is consistent with evidence from *S. aureus, Listeria monocytogenes,* and other Gram-positive species (24, 50–52). Recently, comparative genomic studies in *S. aureus* have linked mutations within the *pde1* ortholog (*gdpP*) to penicillin resistance in natural populations (53–56). Other studies have also linked changes in c-di-AMP concentrations caused by metabolic flux through purine metabolism to penicillin resistance (57). Our work in *S. pneumoniae* extends laboratory findings to natural clinical populations. Surprisingly, despite the diversity and allelic variation of the strains, Pde1 function and its effect on c-di-AMP metabolism were clearly linked to genetic variation in natural populations. Importantly, any exceptions to our main finding that Pde1 loss-of-function increases penicillin resistance can be explained by our mechanistic data, as all exceptions contained *pde1* mutations, many of which we have now confirmed to be loss-of-function in vivo. For example, initially, *pde1* deletion in the clinical isolate Spain^23F^-1 did not increase penicillin resistance. However, with insights from our molecular work, we can now explain this observation. The *pde1* gene in Spain^23F^-1 already contained the A78S and Q339H substitution mutations which we now know to be loss-of-function. Therefore, deletion of the *pde1* gene in Spain^23F^-1 had no effect on the c-di-AMP concentration in the cell, and therefore, no increase in penicillin resistance could be expected if the *pde1* gene was removed in this strain as *pde1* was already functionally inactivated through natural mutation (Fig. 4).

Through a combination of laboratory evolutionary experiments and phylogenetic analyses, our study reveals that rapid de novo *pde1* loss-of-function mutations act as an evolutionary gateway to low-level penicillin resistance. These findings suggest that *pde1* loss-of-function mutations might act as a genetic “scar” for particular *S. pneumoniae* lineages and may explain differences in the evolutionary routes taken by strains as they acquire antibiotic resistance phenotypes and respond to penicillin selection over time.

## Experimental Procedures

### Bacterial Strains and Routine Growth Conditions.

*S. pneumoniae* strains used in this study were derived from D39 Δ*cps* (58) unless otherwise stated. *S. pneumoniae* cells were grown in Todd Hewitt broth containing 0.5% yeast extract at 37 °C with 5% CO_2_. Strains, plasmids, and oligonucleotides used in this study are given in *SI Appendix*, Table S1. The c-di-AMP levels were quantified using a “Cyclic-di-AMP ELISA Kit” from Cayman Chemical (item No. 501960) in accordance with the manufacturer’s instructions. More detailed protocols are given in *SI Appendix* file.

### Selection for Low-Level Ampicillin-Resistant Mutants.

To prepare ampicillin slope plates, molten TSA agar was mixed with defibrinated horse blood (5% final concentration). Ampicillin was added to a final concentration of 0.0625 µg mL^−1^ and 15 mL of the mixture was added to a tilted petri dish (3° approx.), creating a sloped bottom layer. Once the bottom layer set, each plate had a 20 mL TSAII 5% SB layer not containing any ampicillin poured over it on a flat surface. This layer covered the slope completely, resulting in an even agar plate while keeping the ≈3° ampicillin gradient intact. For inoculation, *S. pneumoniae* D39 Δ*cps* cells were grown to mid-exponential phase (OD_600_ = 0.2 to 0.8). Cultures were back diluted to an OD_600_ of exactly 0.2 in THY, and 200 µL of the diluted culture was spread on the slope plates using glass beads. Plates were incubated for 2 d at 37 °C in 5% CO_2_. For each plate, a single bacterial colony that grew beyond the minimal inhibitory ampicillin concentration was picked from the slope plates and cultured. The procedure was repeated multiple times to create 20 independently generated isolates before having their genomes sequenced. SNP and indel analysis for spontaneous ampicillin mutants are given in Dataset S1.

### *S. pneumoniae* MLST Typing.

For the determination of sequence types of clinical isolates, the Illumina short reads were assembled into draft assemblies using SPAdes (version 3.15.5, 2) and the ST was determined using the command line MLST (unpublished software https://github.com/tseemann/mlst) which scans contigs for the seven MLST genes found in the pubMLST scheme (33). The *S. pneumoniae* PubMLST core genome MLST scheme was used to generate an alignment of concatenated nucleotide sequences, and this was used to construct a Neighbor-Joining phylogenetic tree. The full resolution of the tree can be found here: https://microreact.org/project/uALbN1eadVEicDivw1xwMA-spneumopde1final.

### Comparative Genomics Analysis for Sequence Variation of Pde1, Pde2, and CdaA Proteins.

Whole-genome sequences and associated penicillin MIC data for 7293 *S. pneumoniae* isolates stored in the PubMLST database were compared using the Genome Comparator plugin (33). Briefly, the software compares genomic sequences using predefined loci in the database. Each locus is designated a PubMLST allele number based on whether the sequence has previously been identified at that locus. These were exported from the PubMLST database and multiple sequence alignments were produced from the nucleotide sequences using HMMER v.3.2.1. The profile-HMM for each locus was used with the HMMER nucleotide homology search tool, NHMMER, to identify “New” sequences within the *S. pneumoniae* genomes. Custom Python scripts were written to parse the NHMMER output files, including extracting the sequences identified by NHMMER from the isolate assembly files and translating the sequences to amino acid sequences for use in downstream analyses. Multiple sequence alignments of the translated sequences were produced using Clustal Omega and these were used to identify sequence variation from the consensus sequence at each locus. The isolates were separated into groups based on their penicillin resistance profile according to EUCAST breakpoints. For a comparison of total sequence variation of Pde1, Pde2, and CdaA, all variants found within the dataset were normalized by the number of protein sequences available for each group and adjusted for protein length. To visualize the variation at each amino acid locus, the variant frequency was plotted against the amino acid sequence.

## Supplementary Material

Appendix 01 (PDF)

Dataset S01 (XLSX)

## Data Availability

All study data are included in the article and/or supporting information.
