# Supplementary material for: Loss of Pde1 function acts as an evolutionary gateway to penicillin resistance in Streptococcus pneumoniae
Source: Proc Natl Acad Sci U S A. 2023 Oct 5;120(41):e2308029120. doi: 10.1073/pnas.2308029120 (PMC10576035; doi:10.1073/pnas.2308029120)
Supplement: Supplementary file 1 — Appendix 01 (PDF) [file pnas.2308029120.sapp.pdf]

PNAS

Supporting Information for

Loss of Pde1 function acts as an evolutionary gateway to penicillin resistance in *Streptococcus pneumoniae*

Carolin M. Kobras, William Monteith, Sophie Somerville, James M. Delaney, Imran Khan, Camilla Brimble, Rebecca M. Corrigan, Samuel K. Sheppard, Andrew K. Fenton

Andrew K. Fenton
Email: a.k.fenton@sheffield.ac.uk

This PDF file includes:

Supporting text - Supplementary Experimental Procedures
Figures S1 to S13
Table S1
SI References

Other supporting materials for this manuscript include the following:

Datasets S1_Strain genome analysis.xlsx

Supplementary Experimental Procedures

Bacterial strains and routine growth conditions.

All strains used in this study are given in Table S1. All *Streptococcus pneumoniae* strains used in this study were derived from D39 Δ*cps* (1), unless otherwise stated. *S. pneumoniae* strains were routinely grown in Todd Hewitt broth containing 0.5% yeast extract (THY, BD Biosciences) at 37°C in an atmosphere containing 5% CO_2. For growth on solid media, pre-poured Tryptic Soy Agar 5% sheep blood plates were used (TSAII 5% SB; BD Biosciences). Where additives were required, a 5 ml overlay of 1% Nutrient Broth (NB) agar containing these additives were poured over the TSAII 5% SB plates. For antibiotic marker selection, the following concentrations were used: spectinomycin: 100 - 200 µg ml^{-1}, kanamycin: 250 µg ml^{-1}, tetracycline: 0.2 µg ml^{-1}, chloramphenicol: 5 µg ml^{-1}, erythromycin: 0.2 µg ml^{-1}. For induction of gene expression under the P_{fucose} promotor, 0.2% fucose was added to liquid and solid growth media. When finer control of media components was required, TSA plates containing 5% defibrinated horse blood were used, each containing the antibiotics within the plate. *E. coli* strains were routinely grown at 37°C with agitation in LB (1% tryptone, 0.5% yeast extract, 0.17 M NaCl). Solid *E. coli* media contained 1.5% (w/v) agar and selective media contained ampicillin (100 µg ml^{-1}). Bacterial growth was routinely monitored as optical density at a wavelength of 600 nm (OD_{600}) measured spectrophotometrically in 1 cm light path cuvettes.

Selection for low-level ampicillin resistant mutants.

To prepare ampicillin slope plates, molten TSA agar was mixed with defibrinated horse blood (5% final concentration). Ampicillin was added to a final concentration of 0.0625 µg ml^{-1} and 15 ml of the mixture were added to a tilted petri dish (3° approx.), creating a sloped bottom layer. Once the bottom layer set, each plate had a 20 ml TSAII 5% SB layer not containing any ampicillin poured over it on a flat surface. This layer covered the slope completely, resulting in an even agar plate while keeping the $\approx 3^o$ ampicillin gradient intact. For inoculation, *S. pneumoniae* D39 Δ*cps* cells were grown to mid-exponential phase (OD_{600} = 0.2-0.8). Cultures were back diluted to an OD_{600} of exactly 0.2 in THY and 200 µl of the diluted culture were spread on the slope plates using glass beads. Plates were incubated for two days at 37°C in 5% CO_2. For each plate, a single bacterial colony that grew beyond the minimal inhibitory ampicillin concentration was picked from the slope plates and cultured. The procedure was repeated multiple times to create 20 independently generated isolates (Sp_2167, Sp_2169-76, Sp_3073-76, Sp_4131-34, Sp_4136-8).

Genomic DNA preparation

Genomic DNA was prepared using phenol-chloroform extraction. For this, *S. pneumoniae* strains were grown in 20 ml THY to an OD_{600} between 0.2 and 0.8 and cultures were harvested by centrifugation (10 min, 5000 × *g*). Pellets were stored at -20°C until further use. Thawed pellets were combined with 500 µl lysis buffer (20 mM Tris-HCl [pH 7.5], 50 mM EDTA, 100 mM NaCl) and 50 µl lysozyme

(20 mg ml^{-1}) and incubated at 37°C for 1 hour. After incubation, 60 µl of 10% (w/v) N-lauroylsarkosine sodium salt were added and cells were lysed by mixing vigorously. 600 µl phenol were added and the solution was mixed using a vortex before centrifugation at 16000 × *g* for 5 min. The aqueous phase was transferred into a fresh microcentrifuge tube and mixed with 600 µl phenol:chloroform:isoamyl alcohol (25:24:1, v/v) solution. After another centrifugation step (5 min, 16000 × *g*), the aqueous phase was transferred into a fresh microcentrifuge tube and spun down again. 450 µl of the resulting aqueous phase was carefully removed and combined with 1/10 volume 3M NaOAc [pH 5.2] and at least 2 volumes of cold isopropanol (-20°C). The tube was inverted until precipitated genomic DNA was visible and could be pelleted by centrifugation (1 min, 16000 × *g*). The genomic DNA were washed with 700 µl of 70% EtOH, and again spun down by centrifugation (1 min, 16000 × *g*). All traces of ethanol were removed by air-drying the genomic DNA pellet, before re-suspending the pellet in TE buffer (100 mM Tris-HCl [pH 8.0], 1 mM EDTA) containing RNaseA enzyme (≈13 µg ml^{-1}). These preparations were stored at 4°C before use.

Whole genome sequencing and variant detection

Whole-genome sequencing (WGS) was performed by MicrobesNG (http://microbesng.com) using their Illumina sequencing platform. For read trimming, mapping, assembly, and variant analysis of each genome the paired reads were imported into the CLC Workbench software (Build number: 20171114113240). Sequences were trimmed (Quality scores limit: 0.05, Maximum

number of ambiguities = 2) and then mapped onto the reference genome (*S. pneumoniae D39*, accession number: NC_008533) using the following parameters: Mismatch cost = 2, Insertion cost = 3, Deletion cost = 3, Length fraction = 0.7, Similarity fraction = 0.7, Map Randomly. Following read mapping, variants were detected using the basic variant detection tool. Typical coverage filters for variant detection were set at 90% of all reads at a minimum read depth of 20. Variants identified in our WT D39 Δ*cps* strain were removed from the analysis and the data exported. For *pde1* sequences, the identified single nucleotide variants were each validated by PCR amplification and Sanger sequencing.

To identify large deletions >50 bp in length mapped reads were searched for low coverage regions. Typically a low coverage threshold of 10 was applied, but often identified deletions contained 0 coverage. The expected large deletion of the WT 'Δ*cps*' capsule locus (*cps2A-csp2H*, covering ref ≈314640-322145 bp) was identified in each case. This served as an internal control and was removed from the analysis.

S. pneumoniae **MLST typing**

For determination of sequence types (ST) of clinical isolates, the Illumina short reads were assembled into draft assemblies using SPAdes (version 3.15.5, 2) and then the ST was determined using the command line MLST (unpublished software https://github.com/tseemann/mlst) which scans contigs for the seven MLST genes found in the pubMLST scheme (3, 4).

Strain construction and molecular cloning

All plasmids and primer sequences used in this study are listed in Table S1.

S. pneumoniae deletion and integration strains

All *S. pneumoniae* deletion strains were generated using linear PCR fragments as described previously (5). D39 genome sequence information was retrieved using the *x*BASE database (6, 7). Briefly, two ~1-kb flanking regions of each gene were amplified and an antibiotic resistance marker placed between them using isothermal assembly (8). Assembled PCR products were transformed directly into *S. pneumoniae*. In all cases, deletion primers were given the typical name: "gene-designation"_5FLANK_F/R for 5′ regions and "gene-designation"_3FLANK_F/R for 3′ regions, antibiotic markers were amplified from Δ*bgaA* strains using the AntibioticMarker_F/R primers (strains Sp_0005-9). Extracted gDNA from putative deletion strains was confirmed by diagnostic PCR using the AntibioticMarker_R primer in conjunction with a primer binding ≈100 bp 5′ of the disrupted gene; these primers were given the typical name: "gene-designation"_seq_F. Typically ≈200 ng of gDNA was used to generate multiple gene deletions in the D39 Δ*cps* strain background. However, for clinical isolates 2 µg of genomic DNA from previously created marked gene deletions in *S. pneumoniae* strains D39 Δ*cps* and correct insertion confirmed by streaks on selective media or by diagnostic PCR.

P_{fucose}::*pde1* complementation strains and *pde1* point mutations

For construction of pCMK20501, plasmid pAKF205 (5) was amplified by PCRs using Pfuc_XhoI_R and Pfuc_BamHI_F. The *pde1* open reading frame was amplified from *S. pneumoniae* strains D39 Δ*cps* genomic DNA with *pde1*_XhoI_optRBS_F and *pde1*_ORF_BamHI_R. This generated a ≈20 bp overlap between backbone and insert, which were joined using a NEBuilder® HiFi DNA Assembly Kit. *E. coli* DH5α competent cells were transformed with the assembled plasmid and selected colonies were screened for correct insertion by colony PCR using primer pairs: pLEM019_seq_F and pLEM019_seq_R, or pLEM023_F and pLEM023_R. Next, the plasmid was tested by restriction digest (XhoI, BamHI) before final confirmation of the *pde1* ORF insert by Sanger sequencing.

Single point mutations were introduced into the *pde1* ORF by site-directed mutagenesis. For each new plasmid (pCMK20505-10), the template pCMK20501 was amplified by PCR using overlapping primer pairs that introduce the mutation of interest resulting in a circular product. Generally, these primers were designated *pde1*_'aminoacidchange'_F and *pde1*_'aminoacidchange'_R. Where necessary PCR products were digested with DpnI to decrease the number of false positives caused by the pCMK2051 template plasmid. PCR products were transformed into *E. coli* DH5α competent cells. Resultant candidate plasmids were integrity was tested by restriction digest (XhoI, BamHI) and point mutations in *pde1* were subsequently confirmed by Sanger sequencing the whole *pde1* ORF.

All plasmids were inserted into the *bgaA* ectopic locus of the *S. pneumoniae* Δ*pde1* genome by transformation. Site specific insertion of each construct was confirmed

by PCR using primers *bgaA*_PznDiagnostic_F and pLEM023_F, and by streaking on selective media.

Bacterial transformations

S. pneumoniae transformations were performed as described in (5). Briefly, *S. pneumoniae* strains were grown to mid-exponential phase in THY and diluted to an OD_{600} of 0.03. Competence was induced with 500 pg ml^{-1} competence stimulating peptide (CSP-1), 0.2% BSA and 1 mM $CaCl_2$. Typically, after 15 min, 1 ml of culture was transformed with approximately 200 ng of gDNA or plasmid DNA. Transformants were selected on TSAII overlay plates containing 5 µg ml^{-1} chloramphenicol, 0.2 µg ml^{-1} erythromycin, 250 µg ml^{-1} kanamycin, 200 µg ml^{-1} spectinomycin or 0.2 µg ml^{-1} tetracycline as appropriate. Genomic DNA was prepared for mutant strains and correct insertion of the construct was confirmed by PCR.

E. coli competent cells were thawed on ice, carefully mixed with a ligation preparation or isothermal assembly mix and chilled on ice for 30 min. Competent cells were heat shocked at 37°C for 2 min and chilled on ice for 5 min. 450 µl SOC medium (2% tryptone, 0.5% yeast extract, 10 mM NaCl, 2.5 mM KCl, 10 mM $MgCl_2$, 10 mM $MgSO_4$, and 20 mM glucose) were added and cells were recovered for 1 h at 37°C, before plating on LB agar containing 50 µg ml^{-1} ampicillin.

Spot dilutions for antibiotic resistance testing

For antimicrobial resistance testing at low antibiotic concentrations, spot dilution assays were performed. Inoculated to an OD_{600} ≈ 0.025 from an overnight culture, *S. pneumoniae* strains were grown to mid-exponential phase (OD_{600} = 0.2 - 0.8). Cultures were diluted to an OD_{600} of exactly 0.2 and serially diluted (1:10) to a final dilution of 10^{-5} in THY. 5 µl spots of each dilution were spotted onto a range of fresh TSAII 5% SB plates, containing appropriate antibiotic concentrations. Plates were incubated at 37°C in an atmosphere containing 5% CO_2 for ≈ 24 h.

Etest MIC testing

For additional antimicrobial resistance testing using commercially available Etest strips (Biomérieux), *S. pneumoniae* strains were inoculated to an OD_{600} ≈ 0.025 from an overnight culture and grown to mid-exponential phase (OD_{600} = 0.2 - 0.8). These cultures were diluted to an OD_{600} of exactly 0.2 and 200 µl spread on a TSAII 5% SB plate. Once dried, an antibiotic Etest strip was applied, and the plate incubated at 37°C in an atmosphere containing 5% CO_2 for ≈ 24 h.

Growth curves

Starter cultures were prepared by inoculating 20 ml of THY to an OD_{600} ≈ 0.025 from an overnight culture and resulting *S. pneumoniae* strains were grown to mid-exponential phase (OD_{600} = 0.2-0.8). To begin the growth curve, starter cultures were diluted to an OD_{600} of 0.025 in 20 ml THY, incubated at 37°C in 5% CO_2 and

the OD_{600} was recorded every 25-30 min over 6 -7 hours, or until the strains have reached ‘stationary’ growth phase.

Fluorescence microscopy

For *S. pneumoniae* cell labelling, the fluorescent D-amino acid tetramethylrhodamine (TAMRA) 3-amino-D-alanine (TADA, sourced from Tocris) was used to label nascent *S. pneumoniae* cell wall material similar to previously described methods (9, 10). Mid-exponentially growing *S. pneumoniae* cultures (500 µl) were stained with 50 µM TADA for 5 min at 37°C in a 5% CO_2 atmosphere. Cells were centrifuged at 13000 × g for 1 min, washed twice with 500 µl phosphate-buffered saline (PBS) to remove unincorporated TADA. Cells were immobilized on 2% PBS agarose pads and imaged immediately. Fluorescence microscopy was performed on a Nikon Eclipse T*i* inverted microscope through a Nikon Plan Apo ×100 oil objective (NA 1.4). For fluorescence imaging, a SPECTRA X light engine (Lumencor) was used for excitation in combination with the following filter sets, TADA: Ex:438/24, Em:600–660; Dichroic: 595; Images were acquired with a Andor Zyla 4.2 sCMO (Oxford Instruments) at gain 3 using Nikon Elements Software (version 5.11.01). Typical exposure times were between 100–150 ms for TADA labelled cells. Images were processed in ImageJ (11).

Image Analysis

For cell shape and morphology analysis images were analysed using the ImageJ (11) plug in MicrobeJ (v5.131) (12) *S. pneumoniae* cells were generally defined by

the parameters of area: 0.7-3.5 μm^2, length: 0.8-3.5 µm, width: 0.6-1.2 µm, circularity 0.6-1, sinuosity 0.8-max A.U with an area cut-off = 2000. To ensure the best possible sampling, no single image was sampled more than 200 times. The image stacks analysed contained an even mixture of images from three biological replicates arranged for even sampling across each set. All datasets were manually inspected using the TADA fluorescence signal to ensure that cell meshes had been drawn and aligned appropriately. To standardise the sampling, the first 2000 cells (for –capsule anlysis) and the first 2500 cells (for +capsule analysis) were taken for each experimental condition before analysis. For data visualisation, violin plots and box-plots were generated within the MicrobeJ software and exported. Statistical analyses were applied to the datasets within the MIcrobeJ software, first running a Shapiro-Wilk test for normal distribution, before applying a t-test or MannWhitney test as appropriate. A p-value cut-off of 0.05 was applied as the value of significance.

Preparation of whole-cell lysates for cyclic di-AMP quantification

To prepare whole-cell lysates the desired *S. pneumoniae* strains were grown to an OD_{600} of exactly 0.5. 20 ml of this culture was harvested at 3900 ×*g*. The pellet was resuspended in 1 ml of THY medium and 20 µl serially diluted to 10^{-6} and 100 µl plated on TSA blood agar (in triplicate) to determine the colony forming unit (CFU) count of each sample. To create whole-cell lysates themselves, the remaining cell suspension was spun down again (16000 ×*g*) and resuspended in 500 µl B-PER™ Bacterial Protein Extraction Reagent (Thermo Scientific™). After

15 min incubation at room temperature, cleared lysates were checked for complete lysis and for surviving cells by CFU count and by microscopy. Total protein concentrations were determined using a Bradford assay (Bio-Rad), following the manufacturer's instructions. Protein concentrations were calculated using a BSA standard curve using the GraphPad Prism software (version 9.3.1).

Cyclic di-AMP quantification

The c-di-AMP concentrations in cell lysates were tested using the 'Cyclic-di-AMP ELISA Kit' from Cayman Chemical. ELISAs were performed according to the manufacturer's instructions. Briefly, 96-well plates were prepared and at least three different dilutions of each whole-cell lysate were used, each arranged in a technical duplicate (6 wells total per sample). Alongside the samples, c-di-AMP horseradish peroxidase (HRP) conjugate (Tracer) and a limited amount of a c-di-AMP monoclonal antibody were added and incubated for 2 h at room temperature, shaking at 180 rpm. The Tracer and the native c-di-AMP in the whole-cell lysates compete for antibody binding. The antibody-c-di-AMP complex binds to a secondary antibody, previously attached to the wells. The plate was then washed to remove any unbound reagents and a 3,3',5,5' tetramethylbenzidine (TMB) substrate solution was added to the well, followed by the HRP stop solution. The resulting colorimetric reaction was then measured spectrophotometrically in a plate reader (Hidex Sense, Hidex Oy) at an OD of 450 nm. The c-di-AMP concentrations were calculated using a standard curve. For this work controls and standard curves were prepared as instructed by the manufacturer. A non-linear

sigmoidal four parameter logistic curve was fitted on the standard curve using GraphPad Prism (version 9.3.1). The resulting c-di-AMP quantification data was normalised by the protein concentration of the original lysates. Assays were performed using at least three independent biological replicates, each measured in at least six technical replicates. For statistical analysis, the dataset was analysed using a Shapiro-Wilk test (13) to confirm the data was parametric before carrying out a one-tailed Student t-test (14) and a applying a $p > 0.05$ cut off for statistical significance.

Pde1 Immunoblot analysis

S. pneumoniae cultures were grown to an OD_{600} >0.2 in THY +0.2% fucose at 37°C in a 5% CO_2 environment. Cultures were normalized to an OD_{600} of 0.3 and 3 ml harvested by centrifugation at 13000 ×g for 5 min. Cell extracts were prepared by resuspension of cell pellets in 100 µl lysis buffer (20 mM Tris pH 7.5, 10 mM EDTA, 1 mg ml^{-1} lysozyme, 10 µg ml^{-1} DNase I, 100 µg ml^{-1} RNase A, with 0.25x cOmplete Protease Inhibitor [Roche]) and incubated at 37°C for 10 min, followed by addition of 10 µl 10% Sarcosyl for 5 min to induce cell lysis. 100 µl SDS sample buffer (250 mM Tris pH 6.8, 4% SDS, 20% glycerol, 10 mM EDTA) containing 5% 2-mercaptoethanol was added to each prep and samples were heated for 15 min at 50°C prior to storage at -20°C. For each immunoblot, 20 µl of each whole-cell lysate was run on a 12% SDS PAGE gel (40 mA, 150 min) and transferred onto PVDF membranes (60 min, 100V). Membranes were blocked with 5% skimmed milk in 1x PBS 0.5% Tween20 for 1 h (gently rocking, room temperature).

Membranes were washed three times with 1x PBS, 0.05% Tween20, and incubated with primary antibody overnight gently rocking (4°C, 3% BSA in 1x PBS, 0.05% Tween20, 0.02% sodium azide). For detection of Pde1, a mouse anti-Pde1 antibody (15) was used at a final concentration of 1:1000. The blocked PVDF membranes were washed three times with 1x PBS, 0.05% Tween20. The secondary antibody goat anti-mouse IgG conjugated to horseradish peroxidase (used at 1:5000, Biorad Cat #172-1011) was applied to the membrane in 1x PBS with 3% BSA for 1 h at room temperature with gentle shaking. Resulting blots were washed four times in 1x PBS, 0.05% Tween20 and visualised using the Clarity Western ECL Substrate kit (BioRad Cat #170-5061) according to the manufacturer's instructions. Blots were imaged using a Syngene G:BOX Chemi XX9 image quantification device combined with the GeneSys software (v1.6.7.0) using the chemiluminescent detection tools. For quantification of Pde1 bands, the 'quick quantification' tools in the GeneSys software was used and Pde1 bands of interest were selected the band intensity values recorded. These values were normalised to the expression level of the Δ*pde1* P_{fucose}::*pde1* 'wt' strain. Final expression values were averaged across three western blots each using a separately prepared series of whole-cell lysates (n = 3).

Pde1 transformation efficiency assays

For these experiments the Chromosomal Expression Platform (CEP) site was used as a neutral site for kanamycin resistance cassette integration into the *S. pneumoniae* genome (16). Tested strains were grown to an OD_{600} > 0.2 in 5 ml

THY containing 0.2% fucose at 37ºC in a 5% CO_2 environment. Natural competence was induced using CSP-1 as described in the bacterial transformation protocol. 1 ml of resulting competent cells were transformed with 700 ng of CEP::*kan* gDNA (strain Sp_0280) and 100 µl plated on TSAII overlay plates containing 250 µg ml^{-1} kanamycin. These transformations typically gave 500-100 CFU per plate. The number of transformants per experiment was calculated and normalised to the +ve control (strain Sp_4342 Δ*pde1* P_{fuc}::*pde1*). For statistical analysis a one-tailed Student t-test (14) was carried out, applying a $p > 0.05$ cut off for statistical significance.

Comparative genomics analysis for sequence variation of Pde1, Pde2 and CdaA proteins

Whole genome sequences (WGS) and associated penicillin MIC data for 7293 *S. pneumoniae* isolates stored in the PubMLST database were compared using the Genome Comparator plugin (4). Briefly, the software compares genomic sequences using predefined loci in the database. Each locus is designated an allele number based on whether the sequence has previously been identified at that locus. Loci were identified by the Genome Comparator using a minimum identity of 60% across at least 50% of the sequence length. The PubMLST allele numbers have been used where these have been defined, otherwise sequences have been assigned a ‘New’ allele number. Missing and incomplete alleles have been assigned ‘X’ and ‘I’, respectively.

The defined alleles identified by the Genome Comparator at each locus were exported from the PubMLST database. Multiple sequence alignments were produced from the nucleotide sequences using Clustal Omega (17) and a profile-HMM was constructed for each locus using HMMER v.3.2.1 (18). The profile-HMM for each locus was used with the HMMER nucleotide homology search tool, NHMMER (19), to identify 'New' sequences within the *S. pneumoniae* genomes. Custom python scripts were written to parse the NHMMER output files, including extracting the sequences identified by NHMMER from the isolate assembly files and translating the sequences to amino acid sequences for use in downstream analyses. Multiple sequence alignments of the translated sequences were produced using Clustal Omega (17) and these were used to identify sequence variation from the consensus sequence at each locus.

The isolates were separated into groups based on their penicillin resistance profile according to EUCAST breakpoints. 'Resistant' was used for strains with a reported MIC of > 2 mg ml^{-1}, 'intermediate' for 2 mg ml^{-1} ≥ MIC > 0.06 mg ml^{-1}, and 'susceptible' for MICs ≤ 0.06 mg ml^{-1} penicillin. For a comparison of total sequence variation of Pde1, Pde2 and CdaA, all variants found within the dataset were normalised by the number of protein sequences available for each group: [Pde1 R: 1062, I: 2925, S: 3292] [Pde2 R: 1053, I: 2921, S: 3282] [CdaA R: 1038, I: 2911, S: 3286] and adjusted for protein length. To visualise the variation at each amino acid locus, the variant frequency was plotted against the amino acid sequence of Pde1, Pde2 and CdaA respectively. Nonsense-mutations were displayed separately from other nonsynonymous mutations.

To investigate the clonal inheritance of variation identified within Pde1 the phylogenetic ancestry of the isolates was reconstructed using the Interactive tree of life (iTOL) tool (20). The *S. pneumoniae* PubMLST core genome MLST scheme was used to generate an alignment of concatenated nucleotide sequences, and this was used to construct a Neighbor-Joining (NJ) phylogenetic tree.

The full resolution of the tree can be found here:

https://microreact.org/project/uALbN1eadVEicDivw1xwMA-spneumopde1final

To demonstrate the independent emergence of the resistance associated mutations across the phylogeny the ancestral character states at the four mutation sites were reconstructed using the R package, Phangorn (21). The most-parsimonious reconstruction (MPR) attempts to predict the ancestral states within a phylogeny that minimises the total number of character state changes that are required to describe the states observed at the tips of the phylogeny. The MPR for the four mutation sites was predicted using the accelerated reconstruction method (ACCTRAN) (22) and are shown in Figure S8 A-D. The parsimony score, that is the minimum number of changes required to describe the data for a given phylogeny, for the mutations A78S, Q339H, R549C, and T594I are 4145, 4999, 4386, 5351, respectively.

PBP allele diversity measurements

The Genome Comparator plugin in PubMLST (3, 4) was used to identify alleles for the six PBP loci in each *S. pneumoniae* isolate (n = 7169) previously analysed. For each of the four characterised *pde1* mutation hotspots, the isolates were divided

into two groups depending on whether they possess the wild-type residue or variant at that position. The number of unique alleles counted in each group was divided by the group size giving the number of alleles per isolate as a measure of genetic diversity. Incomplete or missing sequences were not included in the analysis.

Figure S1

A

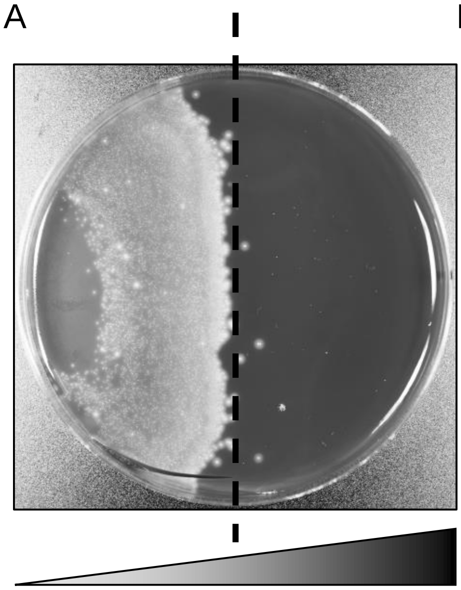

0
62.5
Ampicillin [ng ml^{-1}]

B

No.	Strain	Mosaic PBPs	***murMN*** **or** ***ciaRH*** **resistance determinant**	***pde1*** **ORF Sequence Coverage (%)**	Non-synonymous Mutations	Amino Acid Change
1	Sp2167	No	No	100	146*del*	L49*fs*
2	Sp2169	No	No	100	C → T	Q436*
3	Sp2170	No	No	100	-	-
4	Sp2171	No	No	100	C → T	Q447*
5	Sp2172	No	No	100	-	-
6	Sp2173	No	No	100	-	-
7	Sp2174	No	No	100	-	-
8	Sp2175	No	No	100	C → T	H345Y
9	Sp2176	No	No	100	291+1*ins*	E98*
10	Sp3081	No	No	100	-	-
11	Sp3082	No	No	100	146*del*	L49*fs*
12	Sp3083	No	No	100	-	-
13	Sp3084	No	No	100	G → T	E74*
14	Sp4131	No	No	100	326_347*del*	T109*fs*
15	Sp4132	No	No	100	-	-
16	Sp4133	No	No	100	-	-
17	Sp4134	No	No	100	-	-
18	Sp4136	No	No	100	-	-
19	Sp4137	No	No	100	-	-
20	Sp4138	No	No	100	-	-

Figure S1: *S. pneumoniae* strains selected for low-level ampicillin resistance frequently contain mutations in *pde1*.

(A) A representative example of the ampicillin gradient plates used for selection of low-level *S. pneumoniae* resistant strains. D39 Δ*cps* cells were grown to mid-exponential phase, diluted to an OD_{600} of 0.2 and 200 µl of this culture was plated on TSA plates containing 5% horse blood and an ampicillin gradient ranging from 0 to 62.5 ng ml^{-1}. Plates were incubated for 40-48 h at 37°C in 5% CO_2. For each plate, a single colony that had grown furthest past the approximate zone of inhibition (dashed line) was picked and frozen stocked. Isolated strains were tested for increased ampicillin resistance and their genomic DNA prepared for sequencing. Ampicillin selection was performed in at least 3 independent rounds across a total of 20 plates (n = 20).

(B) Nonsynonymous mutations identified in the *pde1* coding region of low-level ampicillin resistance strains were found in 8 of the 20 isolates (~40%). Coverage = 100% where the whole *pde1* coding region was sequenced by whole-genome sequencing or Sanger sequencing. All putative mutations were confirmed by additional Sanger sequencing from an independent DNA template. *del*: deletion, *ins*: insertion, *fs*: frame-shift, *: stop codon. Amino acid substitutions are indicated by single-letter code. The number of nonsense and missense mutations identified in these strains strongly suggests loss of *pde1* function results in low-level ampicillin resistance. No changes were detected in the '*pbp*', *murMN* and *ciaRH* genes in all strains. The full SNP and indel analysis for these strains are given supplementary Dataset S1.

Figure S2

Growth and morphology measurements of Δ*pde1* and Δ*pde2* strains without capsule

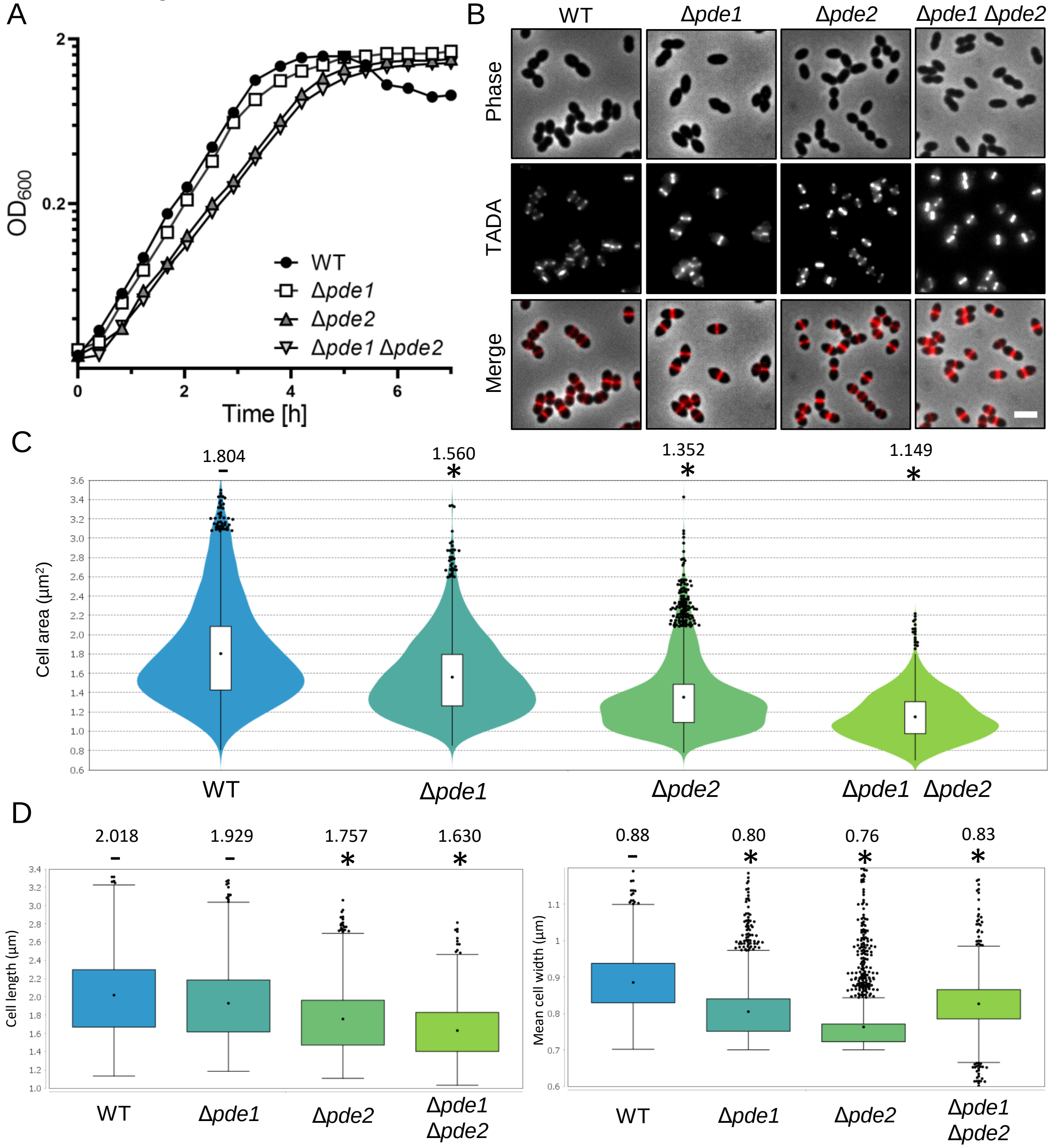

Figure S2: Loss of *pde1* function does not affect cell morphology or growth rate, whereas loss of *pde2* does.

(A) Deletion of *pde1* has no significant effect on growth rate, whereas deletion of *pde2* results in slower growth. Strains were grown to mid-exponential phase, diluted to an OD_{600} of 0.025 and OD_{600} measurements were taken every ~25 min until all strains reached stationary phase. The growth curves are representative for $n \geq 3$ independent repeats. Note, a *pde1 pde2* double mutant shows a growth defect similar to a *pde2* single mutant.

(B) Loss of Pde1 or Pde2 show similar cell shape. Live-cell microscopy data for *pde1* and *pde2* deletion mutants. Strains were grown to mid-exponential phase and labelled with TADA for 5 min before imaging on 2% agarose pads. Scale bar: 2 µm. $n \geq 3$.

(C) Loss of Pde2 function results in a reduction in cell size. Violin plot of cell areas were calculated from cell meshes generated in MicrobeJ. Boxes = +/- QR, spot = mean, dots = outliers.

(D) Box and whisker plots of the length and width measurements of the dataset shown in (C). Boxes = +/- QR, spot = mean, dots = outliers. For the dataset for C and D each strain contains 2500 cell measurements in total, n = 3. Data shows a significant reduction in cell area of Δ*pde2* and Δ*pde1* Δ*pde2* strains compared to WT. Loss of cell area is due to reduction in both length and width. For **C** and **D**, mean values are given above each plot. All statistical test compared distributions to WT, * = p>0.05, NS = not significant.

Figure S3

Growth and morphology measurements of Δ*pde1* and Δ*pde2* strains with capsule

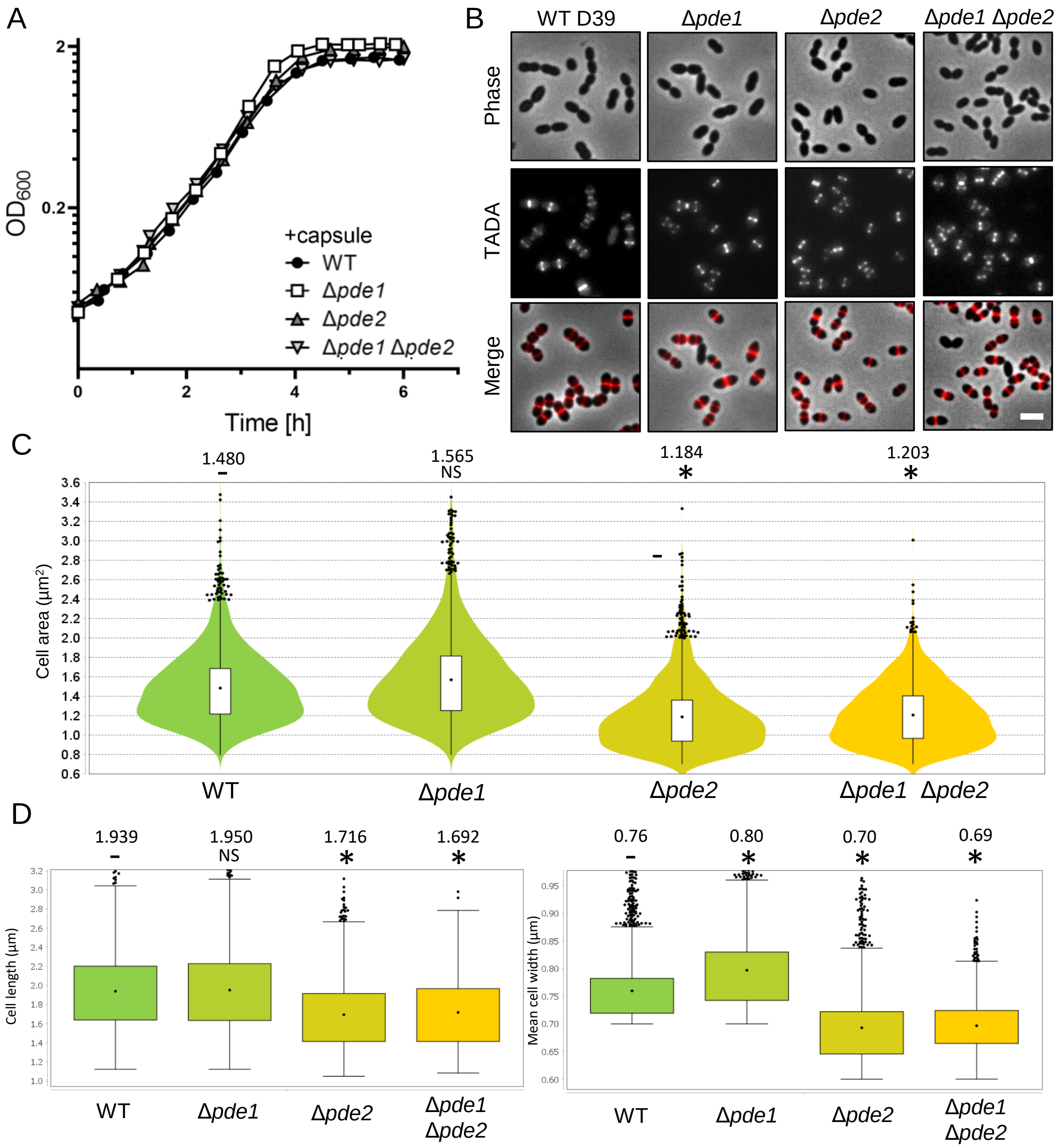

Figure S3: Loss of *pde1* function does not effect cell morphology or growth rate, whereas loss of *pde2* does.

(A) Deletion of *pde1* has no significant effect on growth rate, whereas deletion of *pde2* results in slower growth. Strains were grown to mid-exponential phase, diluted to an OD_{600} of 0.025 and OD_{600} measurements were taken every ~25 min until all strains reached stationary phase. The growth curves are representative for $n \geq 3$ independent repeats. Note, a *pde1* or *pde2* double mutant shows a growth defect similar to a *pde2* single mutant.

(B) Loss of Pde1 or Pde2 show similar cell shape. Live-cell microscopy data for *pde1* and *pde2* deletion mutants. Strains were grown to mid-exponential phase and labelled with TADA for 5 min before imaging on 2% agarose pads. Scale bar: 2 µm. $n \geq 3$.

(C) Loss of Pde2 function results in a reduction in cell size. Violin plot of cell areas were calculated from cell meshes generated in MicrobeJ. Boxes = +/- QR, spot = mean, dots = outliers.

(D) Box and whisker plots of the length and width measurements of the dataset shown in (C). Boxes = +/- QR, spot = mean, dots = outliers. For the dataset for C and D each strain contains 2500 cell measurements in total, n = 3. Data shows a significant reduction in cell area of Δ*pde2* and Δ*pde1* Δ*pde2* strains compared to WT. Loss of cell area is due to reduction in both length and width. For **C** and **D**, mean values are given above each plot. All statistical test compared distributions to WT, * = p>0.05, NS = not significant.

Figure S4

A

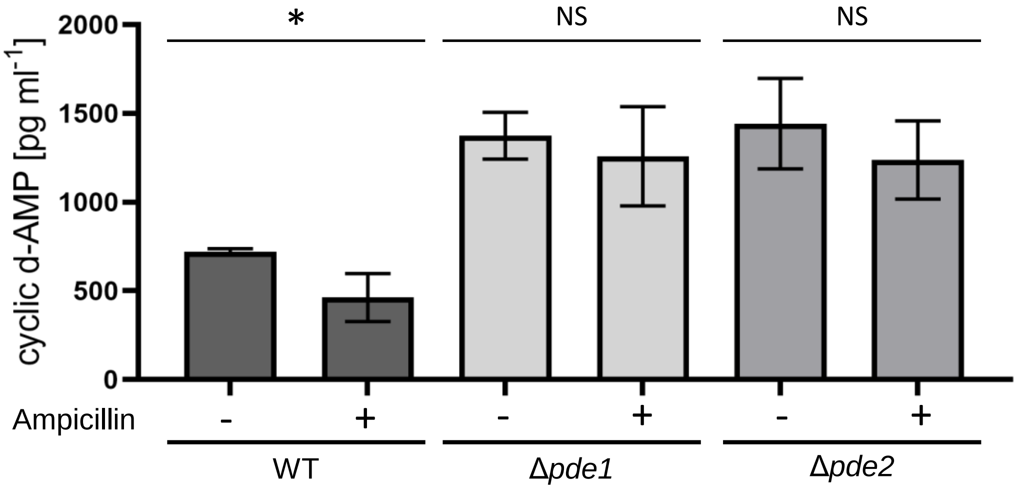

B

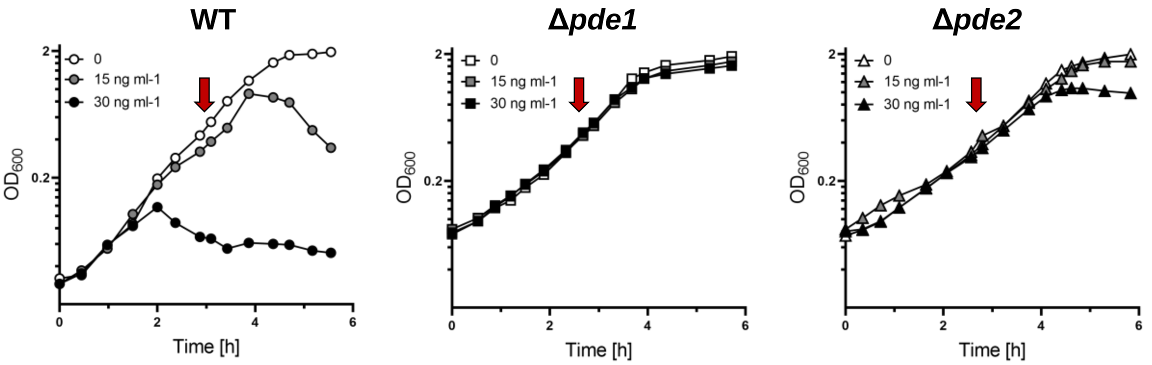

Figure S4: Challenging *S. pneumoniae* with *s*ub-MIC concentrations of ampicillin reduces c-di-AMP concentrations in WT strains

(A) Cyclic-di-AMP quantification in *S. pneumoniae* at ½ MIC concentration of ampicillin. Strains grown in the presence of 15 ng ml^{-1} ampicillin as this represents ½ MIC for WT in THY liquid culture. Plot shows the data from Figure 1E before normalisation. n = 3. * = $p>0.05$, NS = no significant change.

(B) Representative sampling scheme used for the quantitative c-di-AMP measurements shown in A**.** Strains were grown to mid-exponential phase, diluted to an OD_{600} of ≈0.025 in the presence of 30 and 15 ng/ml ampicillin. OD_{600} measurements were taken every ~25 min for 6 h. Cells were removed for c-di-AMP quantification when cultures reached an OD_{600} of 0.5 (red arrow). Representative growth curve and sampling shown, n = 3.

Figure S5

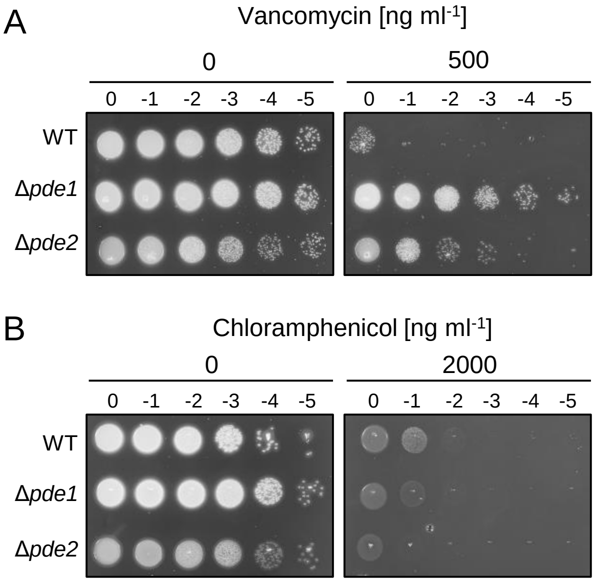

C

Strain	Pen MIC [ng ml^{-1}]	Van MIC [ng ml^{-1}]	Erm MIC [ng ml^{-1}]	Chl MIC [ng ml^{-1}]
wt	6	380	640	1500
Δ*pde1*	12	500	640	1500
Δ*pde2*	8	500	640	1500

Figure S5: Loss of *pde1* or *pde2* function increase resistance to cell wall synthesis-targeting antibiotics.

Spot dilution of wild type D39 Δ*cps* and its derivatives on plates containing antibiotics as indicated. Strains were grown to mid-exponential phase, diluted to an OD_{600} of 0.2 (0) and further serially diluted to 10^{-5} (-5). 5 µl of each dilution were spotted onto TSA plates containing 5% horse blood and the antibiotic concentration indicated in the figure. Plates were incubated for 20-24 h at 37°C in 5% CO_2. The displayed plates are representative for $n \geq 4$ biological repeats with the same effect.

(A) Spot dilutions indicate Pde1 or Pde2 loss of function increases resistance to the cell wall targeting glycopeptide antibiotic vancomycin compared to WT. Note, as for ampicillin (Fig. 1), the effect of *pde2* deletion on viability counts were less than the *pde1* deletion strain.

(B) Spot dilutions indicate Pde1 or Pde2 loss of function does not change the sensitivity to the protein biosynthesis-targeting antibiotic chloramphenicol when compared to WT.

(C) Etest MIC data for each strain, Pen = PenicillinG, Van = vancomycin, Erm = erythromycin, Chl = chloramphenicol.

Figure S6

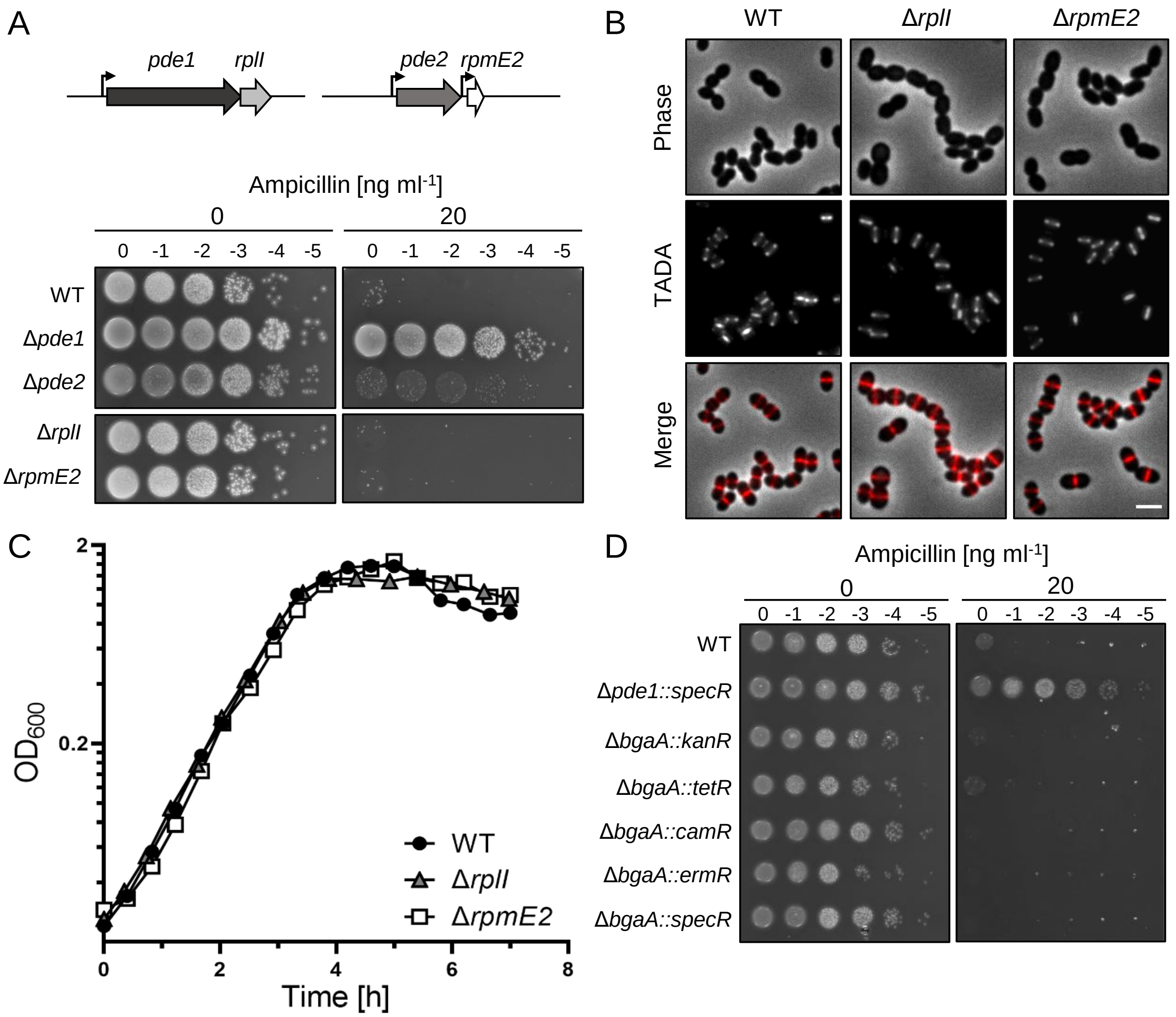

Figure S6: The increase in ampicillin resistance upon *pde1* or *pde2* loss of function is not due to non-specific genetic effects.

(A) The *pde1* and *pde2* ampicillin resistance phenotype is not a result of genetic polar effects on *rplI* and *rpmE2* open reading frames*.* The schematic displays the *pde1* and *pde2* loci with adjacent genes, respectively. Grey and white arrows display coding sequences of the genes indicated above and black arrows indicate putative promoter regions. For all spot dilutions, strains were grown to mid-exponential phase, diluted to an OD_{600} of 0.2 (0) and further serially diluted to 10^{-5} (-5). 5 µl of each dilution were spotted onto TSA plates containing 5% horse blood and the antibiotic concentration indicated in the figure. Plates were incubated for 20-24 h at 37°C in 5% CO_2. The displayed plates are representative for $n \geq 4$ biological repeats with the same effect.

(B) Deletion of *rplI* and *rpmE2* does not affect cell morphology. Cells were grown to mid-exponential phase and labelled with TADA for 5 min before imaging on 2% agarose pads. Scale bar: 2 µm. $n \geq 3$.

(C) Deletion of *rplI* and *rpmE2* does not affect growth. Strains were grown to mid-exponential phase, diluted to an OD_{600} of 0.025 and OD_{600} measurements were taken every ~25 min until all strains reached stationary growth phase. The growth curves are representative for $n \geq 3$ independent repeats.

(D) The introduction of antibiotic markers into the *S. pneumoniae* genome does not affect ampicillin resistance. Strains containing all antibiotic markers used in this study were inserted into the *bgaA* ectopic locus. In all cases these insertions did not impact ampicillin resistance when compared to *pde1* loss of function strains.

Figure S7

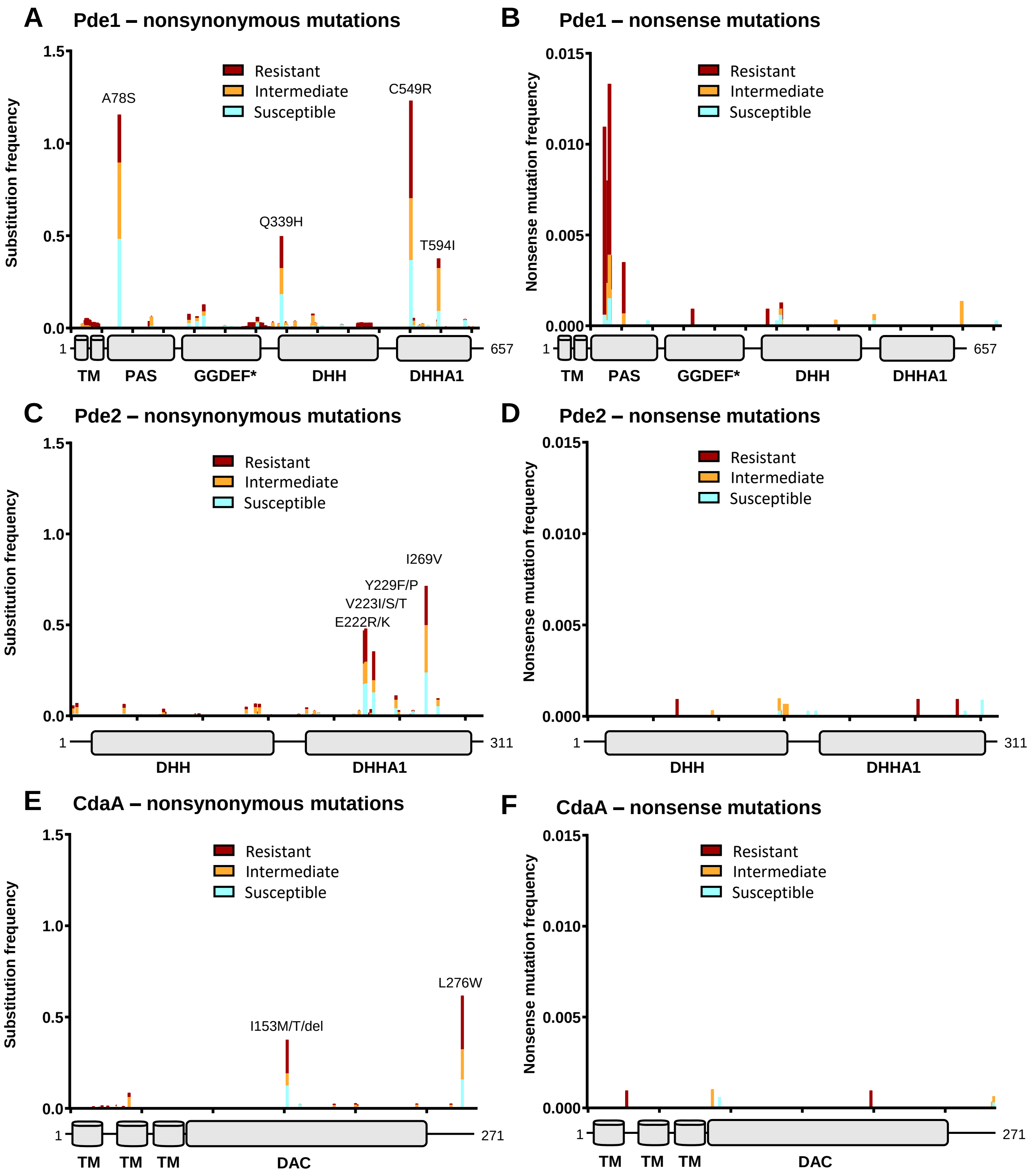

A
Pde1 – nonsynonymous mutations
Substitution frequency
1.5
1.0
0.5
0.0
Resistant
Intermediate
Susceptible
A78S
Q339H
C549R
T594I
1
657
TM
PAS
GGDEF*
DHH
DHHA1
B
Pde1 – nonsense mutations
Nonsense mutation frequency
0.015
0.010
0.005
0.000
Resistant
Intermediate
Susceptible
1
657
TM
PAS
GGDEF*
DHH
DHHA1
C
Pde2 – nonsynonymous mutations
Substitution frequency
1.5
1.0
0.5
0.0
Resistant
Intermediate
Susceptible
I269V
Y229F/P
V223I/S/T
E222R/K
1
311
DHH
DHHA1
D
Pde2 – nonsense mutations
Nonsense mutation frequency
0.015
0.010
0.005
0.000
Resistant
Intermediate
Susceptible
1
311
DHH
DHHA1
E
CdaA – nonsynonymous mutations
Substitution frequency
1.5
1.0
0.5
0.0
Resistant
Intermediate
Susceptible
L276W
I153M/T/del
1
271
TM
TM
TM
DAC
F
CdaA – nonsense mutations
Nonsense mutation frequency
0.015
0.010
0.005
0.000
Resistant
Intermediate
Susceptible
1
271
TM
TM
TM
DAC

Figure legend for Figure S7

Figure S7 : Genetic variation of Pde1 is increased in resistant *S. pneumoniae* isolates.

Phylogenetic data comparing sequence variation in Pde1 to genes and protein sequences found in the same pathway: Pde2 and CdaA. This acts as a comparison figure for the data show in Fig. 3A and Fig. 3B. Data for Pde1 is duplicated from Fig. 3C + Fig. 3D for clarity and easier comparison. Genome sequences of >7200 *S. pneumoniae* isolates were retrieved from the PubMLST data base (4), alongside their known MIC for penicillin. Isolates were split into groups based on their penicillin resistance profile according to EUCAST breakpoints. 'Resistant' was used for strains with a reported MIC of > 2 mg ml^{-1} (dark red), 'intermediate' for 2 mg ml^{-1} ≥ MIC > 0.06 mg ml^{-1} (red), and 'susceptible' for MICs ≤ 0.06 mg ml^{-1} penicillin (cyan). For A and B, all mutations found within the dataset were normalised by the number of protein sequences available for each group: [Pde1 R: 1062, I: 2925, S: 3292] [Pde2 R: 1053, I: 2921, S: 3282] [CdaA R: 1038, I: 2911, S: 3286] and adjusted for protein length.

(A) Sequence variation for Pde1 at each amino acid position reveals four residues are often mutated in this protein. Together, these mutations account for ≈52% of total Pde1 mutations in the dataset. Amino acid changes are indicated where the mutation frequency is > 0.25.

(B) Pde1 nonsense mutations are more frequent in resistant *S. pneumoniae* isolates compared to intermediate or susceptible strains. Plotting the variation at each amino acid position of Pde1 revealed an accumulation of stop codons in the putative N-terminal transmembrane helices.

(C) Sequence variation in Pde2 is enriched in the DHHA1 enzymatic domain. Amino acid changes are indicated where mutation frequency > 0.25.

(D) *S. pneumoniae* isolates do not display significant numbers of nonsense mutations in *pde2*.

(E) Sequence variation was overall lower in CdaA, compared to Pde1 and Pde2.

(F) Very few nonsense mutations were identified across the *cdaA* coding sequence, stressing the essentiality of CdaA in *S. pneumoniae*. Protein domains are labelled: TM: Transmembrane helix, PAS: Per-Arnt-Sim domain, GGDEF*: atypical GGDEF domain. DHH: DHH phosphatase family domain, and DHHA1: DHH associated domain. DAC: Diadenylate cyclase. Amino acid changes are indicated where mutation frequency > 0.25.

Figure S8

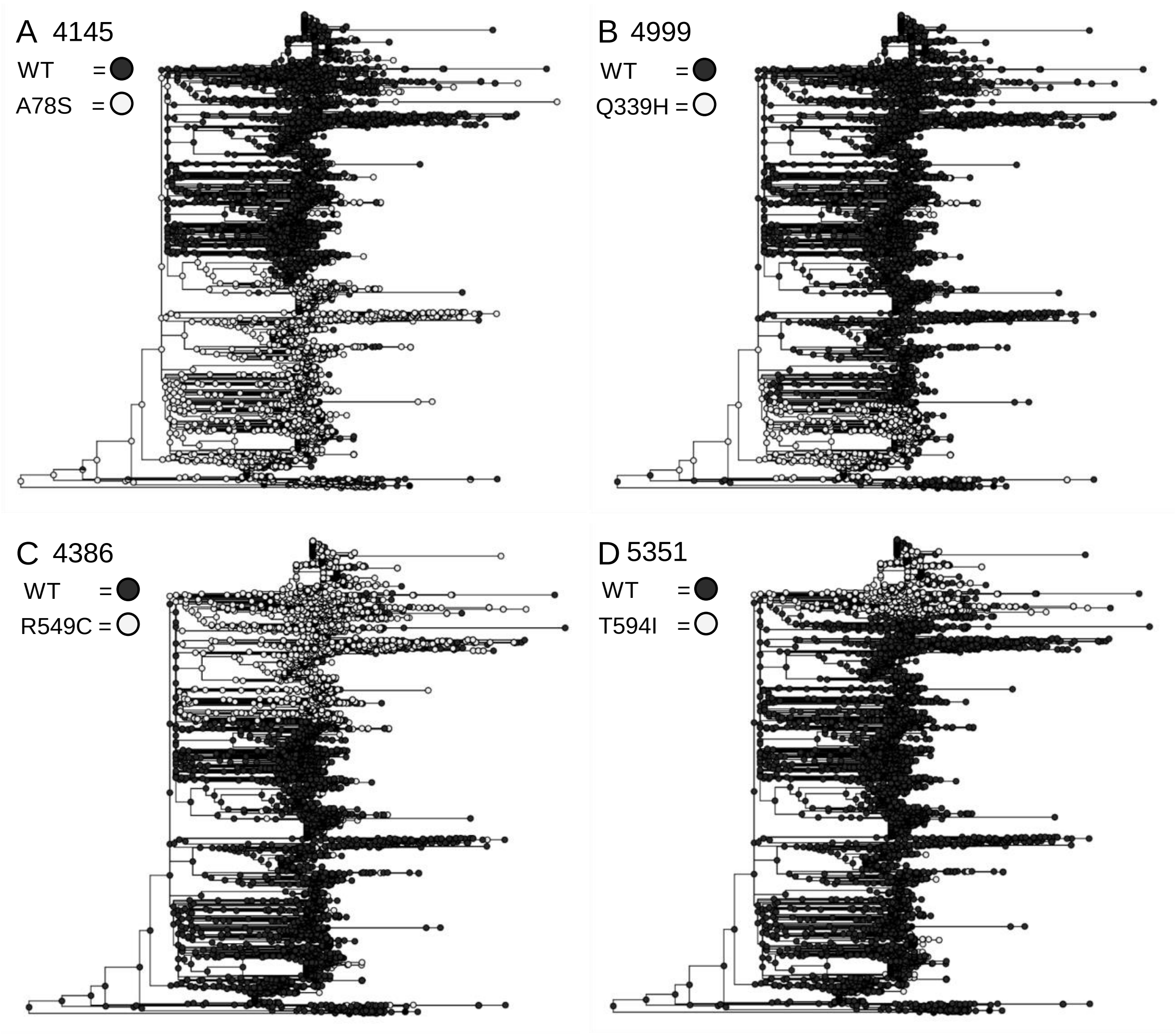

Figure S8. Phylogeny of 7272 *S. pneumoniae* genomes with Pde1 ancestral state reconstruction at common amino acid substitution sites. Nodes representing the wild-type state are shown in black and nodes representing the indicated amino acid substitution are shown in white. **A** shows the A78S substitution, **B** the Q339H, **C** the R549C, and **D** the T594I. The minimum number of changes to fully explain the distribution of the dataset are shown on each phylogeny. In each case, whilst each substitution shows vertical inheritance patterns, the number of substitutions suggests *de novo* or through multiple homologous gene transfer events have occurred.

Figure S9

Group	Strain	ST	MIC	Mosaic PBPs	Pde1 mutations	Δ*pde1* effect on MIC	Pen MIC [ng ml^{-1}] WT	Pen MIC [ng ml^{-1}] Δ*pde1*	Ampicillin [ng ml^{-1}]
Lab strains	**D39 Δ*cps***	595	S	No	*R549C*	Yes	6	12	15
Lab strains	**R6**	595	S	No	*R549C*	Yes	16	23	25
Vaccine serotypes	**Spain[23F]-1**	81	I	Yes	A78S Q339H *R549C*	No	750	750	2000
Vaccine serotypes	**Taiwan[19F]-14**	236	I	Yes	none	Yes	380	750	450
Vaccine serotypes	**Tennessee[14]-18**	67	R	Yes	none	Yes	2000	3000	600
Vaccine serotypes	**Sweden[1]-27**	217	S	No	none	Yes	12	16	20
Prevalent serotypes	**Netherlands[8]-33**	53	S	-	A78S E636K	No	12	16	20
Prevalent serotypes	**MB280669F[9N]**	66	S	No	none	Yes	8	16	20
Prevalent serotypes	**Denmark[12F]-34**	218	S	No	Q339H	No	12	8	30
Prevalent serotypes	**Netherlands[15B]-37**	199	S	-	*R549C*	Yes	6	12	30
Prevalent serotypes	**MB167313[35B]**	198	I	No	*R549C*	Yes	750	1250	300
Prevalent serotypes	**Utah[35B]-24**	377	I	-	*R549C*	Yes	750	1000	4000
Clinical isolates	**MB166525[15A]**	3811	S	No	A189E Y213H	Yes	20	80	20
Clinical isolates	**MB166710[15A]**	63	I	Yes	T594I	Yes	30	47	90

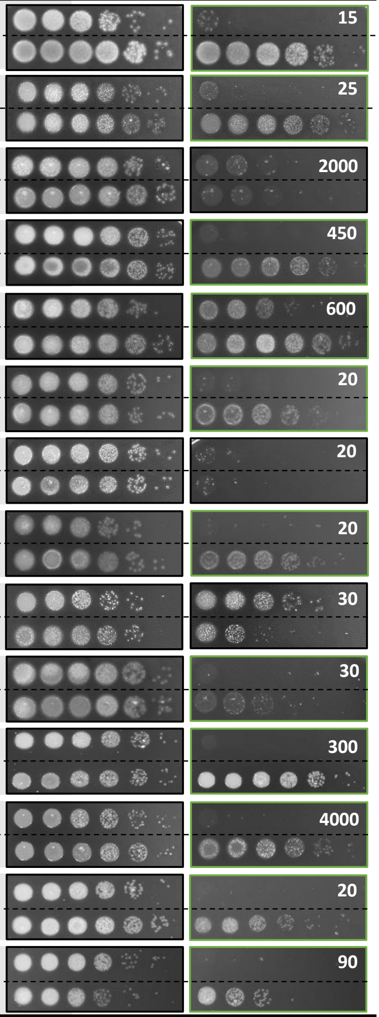

Figure S9: Loss of Pde1 function increases ampicillin resistance in a range of *S. pneumoniae* clinical isolates.

In addition to the laboratory strain D39 Δ*cps* and its derivative R6, *pde1* deletion led to increased ampicillin resistance in clinical strains including serotypes covered by the PCV-13 vaccine, serotypes currently prevalent in the world (post-PCV13), and local clinical isolates. Importantly, the effect could also be observed in isolates classified as intermediate or resistant. Serotypes for each strain are given in superscript. Where the strain is part of the Pneumococcal Molecular Epidemiology Network (PMEN), the number in the collection is indicated by a dash following the strain name. Mosaic PBPs were classified as present where one or more PBP had significant sequence variation (>1%). A dash indicates no information. Nonsynonymous mutations in the Pde1 sequence of clinical strains are indicated by single letter amino acid code. Representative spot plates (n ≥ 2) with an increase in resistance upon *pde1* deletion are framed in green. Importantly where deleting *pde1* did not effect resistance these genomes already contained Pde1 variants which are now known to be loss of function (compare mutations with those tested in Fig. 5).

Figure S10

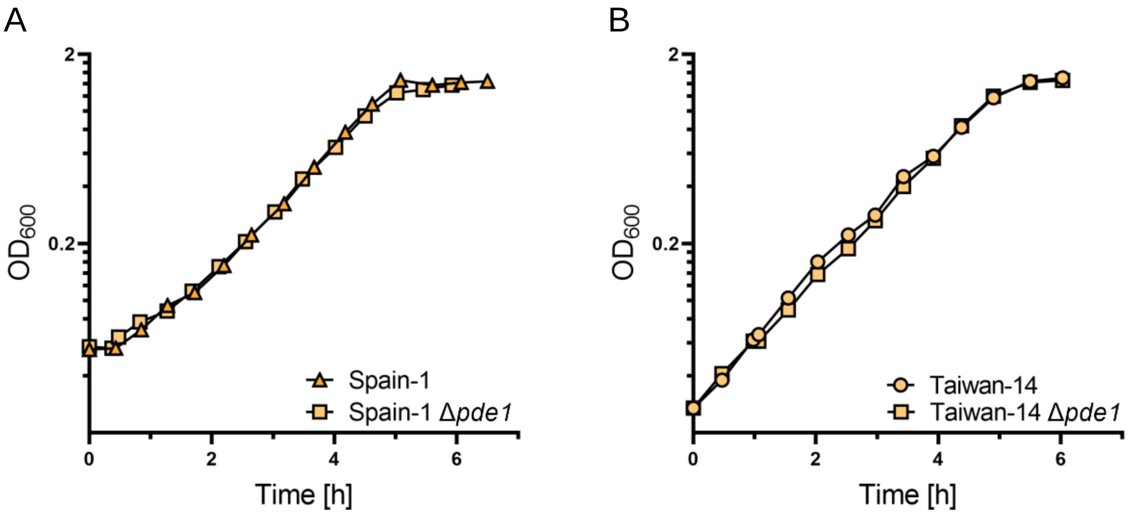

Figure S10: The loss of *pde1* function does not reduce growth rate in the Spain-1 and Taiwan-14 *S.pneumoniae* clinical isolates. Strains were grown to mid-exponential phase, diluted to an OD_{600} of 0.025 and OD_{600} measurements were taken every ~25 min until all strains reached stationary growth phase. The growth curves are representative for n = 3 independent repeats.

Figure S11

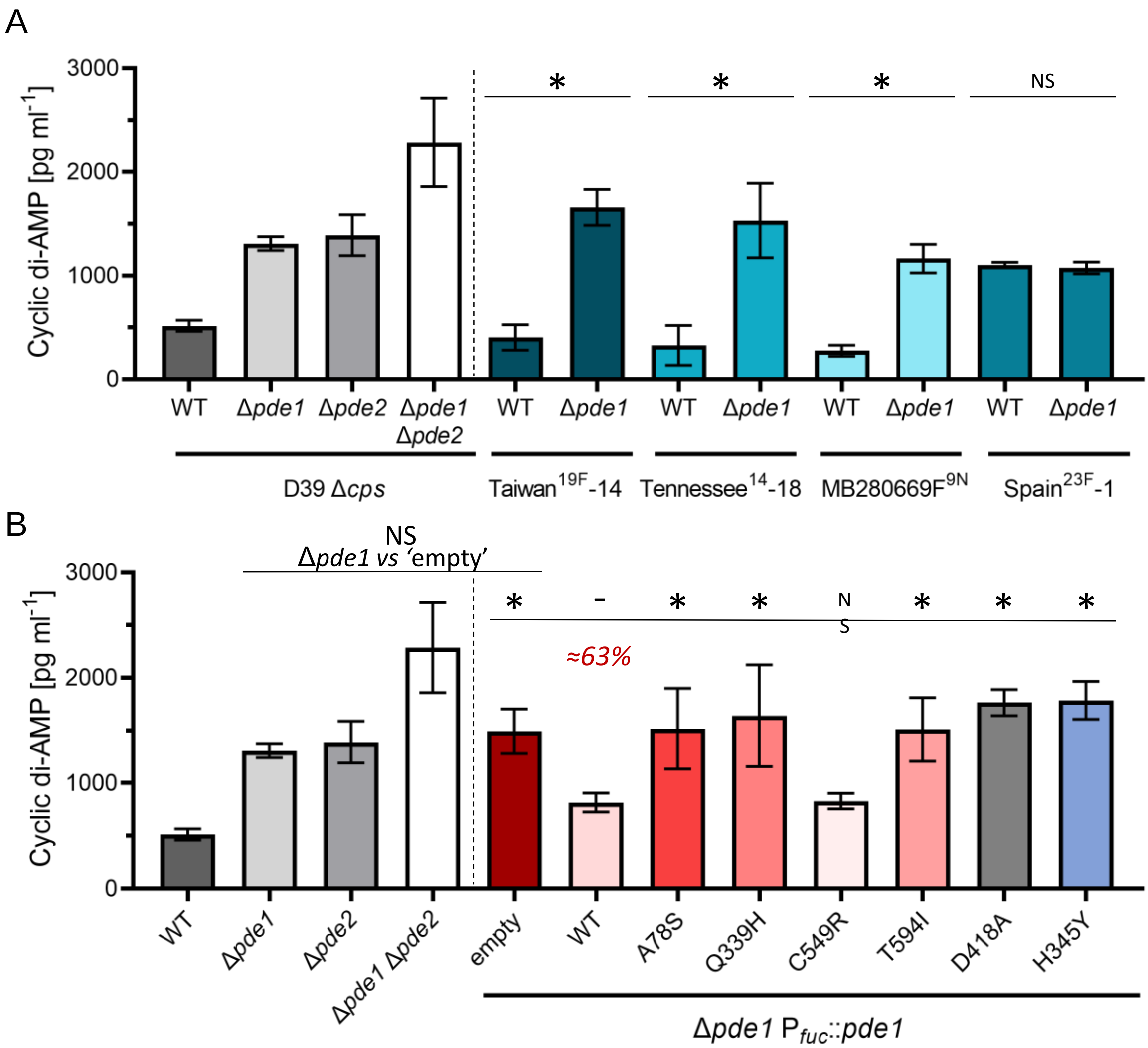

Figure S11: Loss of Pde1 function increases cyclic-di-AMP levels in a range of *S. pneumoniae* clinical isolates.

(A) The c-di-AMP concentration of four clinical isolates, representative for the susceptible, intermediate and resistant category, was elevated in *pde1* deletion strains compared to the WT. Here, data from clinical strains are set in comparison to the deletion strain data shown in Fig. 1. n = 3.

(B) The c-di-AMP concentration of *pde1* complementation strains compared to the deletion strain data shown in Fig. 1.

* denotes a statistical significant change compared to the WT complementation strain (p>0.05) measured using a t-test, NS = not significant, - = the normalised WT dataset. Note, the complementation of Δ*pde1* strains using the P_{fucose}::*pde1* construct results in ≈ 63% restoration of c-di-AMP levels when compared to WT. n = 3.

Figure S12

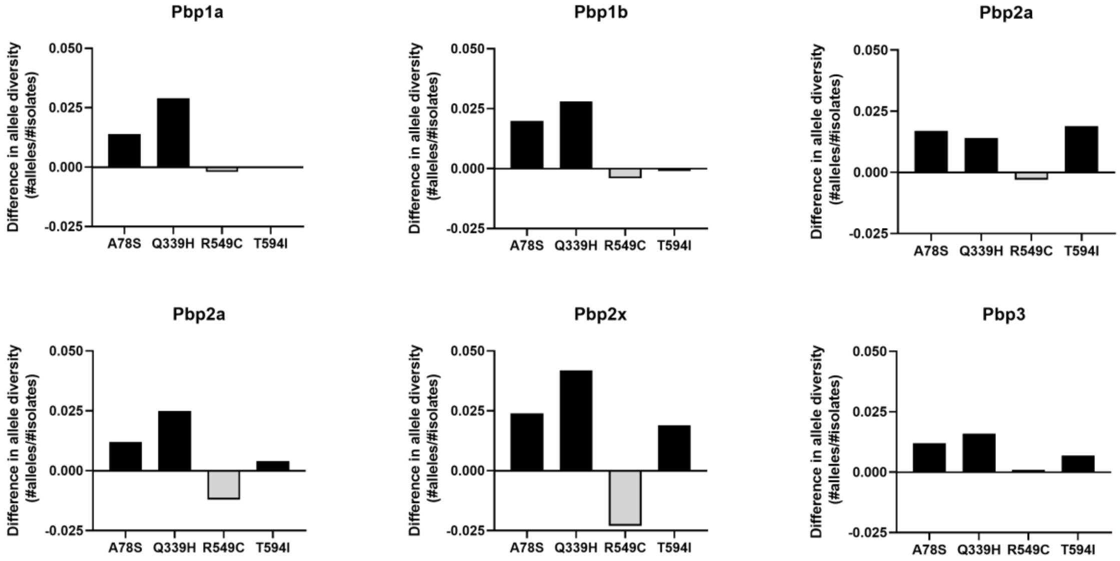

Figure S12: PBP allele diversity is increased in *S. pneumoniae* populations containing Pde1 loss-of-function variants. The data shows higher allele diversity across all *pbp* genes when strains contain a known *pde1* loss-of-function variant(s). Note the R549C variant acts as a control in these plots as it is now known to be fully functional (grey). All loss of function variants have higher degrees of diversity (black) with the greatest change shown for A78S and Q339H. The Genome Comparator was used to identify *pbp* allele number as a measure of sequence diversity. The PBP allele diversity measurements were split by the Pde1 variant. The allele frequency data used to generate these plots are given in Figure S13.

Figure S13

PBP (gene name)	SNP		Number of isolates	Number of alleles	Allele Diversity (Allele number/Isolate number)
PBP1A (*pbp1A*)	A78S	A	4089	212	0.052
		S	3075	203	0.066
	Q339H	Q	5953	296	0.050
		H	1202	95	0.079
	R549C	R	4400	254	0.058
		C	2752	155	0.056
	T594I	T	6112	328	0.054
		I	1029	56	0.054
PBP1b (*pbp1B*)	A78S	A	4089	258	0.063
		S	3075	256	0.083
	Q339H	Q	5953	373	0.063
		H	1202	109	0.091
	R549C	R	4400	316	0.072
		C	2752	188	0.068
	T594I	T	6112	393	0.064
		I	1029	65	0.063
PBP2a (*pbp2A*)	A78S	A	4089	227	0.056
		S	3075	225	0.073
	Q339H	Q	5953	334	0.056
		H	1202	84	0.070
	R549C	R	4400	275	0.063
		C	2752	164	0.060
	T594I	T	6112	334	0.055
		I	1029	76	0.074
PBP2b (*penA*)	A78S	A	4089	364	0.089
		S	3075	311	0.101
	Q339H	Q	5953	522	0.088
		H	1202	136	0.113
	R549C	R	4400	439	0.100
		C	2752	241	0.088
	T594I	T	6112	560	0.092
		I	1029	99	0.096
PBP2x (*pbpX*)	A78S	A	4089	415	0.101
		S	3075	385	0.125
	Q339H	Q	5953	599	0.101
		H	1202	172	0.143
	R549C	R	4400	528	0.120
		C	2752	268	0.097
	T594I	T	6112	624	0.102
		I	1029	125	0.121
PBP3 (*dacC*)	A78S	A	4089	123	0.030
		S	3075	128	0.042
	Q339H	Q	5953	178	0.030
		H	1202	55	0.046
	R549C	R	4400	152	0.035
		C	2752	99	0.036
	T594I	T	6112	190	0.031
		I	1029	39	0.038

Figure S13: PBP allele diversity measurements in *S. pneumoniae* populations containing Pde1 loss-of-function variants. The Genome Comparator was used to identify the number of *pbp* alleles in each group as a measure of sequence diversity. PBP allele diversity measurements were split by the identified Pde1 variant. The highest figures for each allele diversity ratio are highlighted in red when the variant is known loss of function and yellow in the R549C known Pde1^{wt}.

Table S1 Strains, plasmids and oligonucleotides used in this study

S. pneumoniae			
**Strain Name**	**Genotype/Sequence**	**Resistance Marker(s)**	**Source or Reference**
D39 'Sp_0001'	D39 [Type Strain] NCTC 7466	-	(1)
D39 Δ*cps* 'Sp_0002'	D39 Δ*cps2A*'-Δ*cps2H'* [Wild Type]	-	(1)
Sp_2167	D39 Δ*cps* - selected on ampicillin gradient plates [0-0.0625 µg/ml] – Isolate #1	-	This study
Sp_2169	D39 Δ*cps* - selected on ampicillin gradient plates [0-0.0625 µg/ml] – Isolate #2	-	This study
Sp_2170	D39 Δ*cps* - selected on ampicillin gradient plates [0-0.0625 µg/ml] – Isolate #3	-	This study
Sp_2171	D39 Δ*cps* - selected on ampicillin gradient plates [0-0.0625 µg/ml] – Isolate #4	-	This study
Sp_2172	D39 Δ*cps* - selected on ampicillin gradient plates [0-0.0625 µg/ml] – Isolate #5	-	This study
Sp_2173	D39 Δ*cps* - selected on ampicillin gradient plates [0-0.0625 µg/ml] – Isolate #6	-	This study
Sp_2174	D39 Δ*cps* - selected on ampicillin gradient plates [0-0.0625 µg/ml] – Isolate #7	-	This study
Sp_2175	D39 Δ*cps* - selected on ampicillin gradient plates [0-0.0625 µg/ml] – Isolate #8	-	This study
Sp_2176	D39 Δ*cps* - selected on ampicillin gradient plates [0-0.0625 µg/ml] – Isolate #9	-	This study
Sp_3081	D39 Δ*cps* - selected on ampicillin gradient plates [0-0.0625 µg/ml] – Isolate #10	-	This study
Sp_3082	D39 Δ*cps* - selected on ampicillin gradient plates [0-0.0625 µg/ml] – Isolate #11	-	This study
Sp_3083	D39 Δ*cps* - selected on ampicillin gradient plates [0-0.0625 µg/ml] – Isolate #12	-	This study
Sp_3084	D39 Δ*cps* - selected on ampicillin gradient plates [0-0.0625 µg/ml] – Isolate #13	-	This study
Sp_4131	D39 Δ*cps* - selected on ampicillin gradient plates [0-0.0625 µg/ml] – Isolate #14	-	This study
Sp_4132	D39 Δ*cps* - selected on ampicillin gradient plates [0-0.0625 µg/ml] – Isolate #15	-	This study
Sp_4133	D39 Δ*cps* - selected on ampicillin gradient plates [0-0.0625 µg/ml] – Isolate #16	-	This study
Sp_4134	D39 Δ*cps* - selected on ampicillin gradient plates [0-0.0625 µg/ml] – Isolate #17	-	This study
Sp_4136	D39 Δ*cps* - selected on ampicillin gradient plates [0-0.0625 µg/ml] – Isolate #18	-	This study
Sp_4137	D39 Δ*cps* - selected on ampicillin gradient plates [0-0.0625 µg/ml] – Isolate #19	-	This study
Sp_4138	D39 Δ*cps* - selected on ampicillin gradient plates [0-0.0625 µg/ml] – Isolate #20	-	This study
Sp_0005	D39 Δ*cps* Δ*bgaA*::kan	Kan	(5)
Sp_0006	D39 Δ*cps* Δ*bgaA*::*add9*(spec)	Spec	(5)
Sp_0007	D39 Δ*cps* Δ*bgaA*::*tetM*(tet)	Tet	(5)
Sp_0008	D39 Δ*cps* Δ*bgaA*::*cat*(cam)	Cam	(5)
Sp_0009	D39 Δ*cps* Δ*bgaA*::*erm*	Erm	(5)
Sp_2104	D39 Δ*cps* Δ*pde1*::*spec*	Spec	This study
Sp_2121	D39 Δ*cps* Δ*pde1*::spec Δ*pde2*::*kan*	Spec, Kan	This study

Sp_4033	D39 Δ*cps* Δ*rplI*::*spec*	Spec	This study
Sp_4037	D39 Δ*cps* Δ*pde2*::*kan*	Kan	This study
Sp_4039	D39 Δ*cps* Δ*rpmE2*::*spec*	Spec	This study
Sp_0310	D39 (+capsule) Δ*pde1*::*spec*	Spec	This study
Sp_0312	D39 (+capsule) Δ*pde2*::*kan*	Kan	This study
Sp_0314	D39 (+capsule) Δ*pde1*::spec Δ*pde2*::*kan*	Spec, Kan	This study
R6<br>Sp_0003	R6	-	(23)
Sp_4185	R6 Δ*pde1*::*spec*	Spec	This study
Spain^{23F}-1<br>Sp_0022	Spain^{23F}-1	-	(24-26)
Sp_4093	Spain^{23F}-1 Δ*pde1*::*spec*	Spec	This study
Taiwan^{19F}-14<br>Sp_0035	Taiwan^{19F}-14	-	(27)
Sp_4398	Taiwan^{19F}-14 Δ*pde1*::*spec*	Spec	This study
Tennessee^{14}-18<br>Sp_0039	Tennessee^{14}-18	-	(28)
Sp_4396	Tennessee^{14}-18 Δ*pde1*::*spec*	Spec	This study
Utah^{35B}-24<br>Sp_0045	Utah^{35B}-24	-	(29)
Sp_4098	Utah^{35B}-24 Δ*pde1*::*spec*	Spec	This study
Sweden^{1}-27<br>Sp_0130	Sweden^{1}-27	-	(30, 31)
Sp_4101	Sweden^{1}-27 Δ*pde1*::*spec*	Spec	This study
Netherlands^{8}-33<br>Sp_0136	Netherlands^{8}-33	-	(32)
Sp_4105	Netherlands^{8}-33 Δ*pde1*::*spec*	Spec	This study
Denmark^{12F}-34<br>Sp_0137	Denmark^{12F}-34	-	(32)
Sp_4189	Denmark^{12F}-34 Δ*pde1*::*spec*	Spec	This study
Netherlands^{15B}-37<br>Sp_0140	Netherlands^{15B}-37	-	(32)
Sp_4193	Netherlands^{15B}-37 Δ*pde1*::*spec*	Spec	This study
Sp_0151	MB280669F^{9N}	-	Northern General Hospital Sheffield
Sp_4109	MB280669F^{9N} Δ*pde1*::*spec*	Spec	This study
Sp_0066	MB167313^{35B}	-	Northern General Hospital Sheffield
Sp_2181	MB167313^{35B} Δ*pde1*::*spec*	Spec	This study
Sp_0067	MB166525^{15A}	-	Northern General Hospital Sheffield
Sp_2113	MB166525^{15A} Δ*pde1*::*spec*	Spec	This study
Sp_0062	MB166710^{15A}	-	Northern General Hospital Sheffield
Sp_2109	MB166710^{15A} Δ*pde1*::*spec*	Spec	This study
Sp_4330	D39 Δ*cps* Δ*bgaA*::P_{fucose}-*empty-tet*	Tet	This study
Sp_4332	D39 Δ*cps* Δ*bgaA*::P_{fucose}-*pde1-tet*	Tet	This study
Sp_4340	D39 Δ*cps* Δ*pde1*::spec Δ*bgaA*::P_{fucose}-*empty-tet*	Tet, Spec	This study
Sp_4342	D39 Δ*cps* Δ*pde1*::spec Δ*bgaA*::P_{fucose}-*pde1-tet*	Tet, Spec	This study

Sp_4391	D39 Δ*cps* Δ*pde1*::spec Δ*bgaA*::P_{fucose}-*pde1*[A78S]-*tet*	Tet, Spec	This study
Sp_4389	D39 Δ*cps* Δ*pde1*::spec Δ*bgaA*::P_{fucose}-*pde1*[C549R]-*tet*	Tet, Spec	This study
Sp_4422	D39 Δ*cps* Δ*pde1*::spec Δ*bgaA*::P_{fucose}-*pde1*[Q339H]-*tet*	Tet, Spec	This study
Sp_4424	D39 Δ*cps* Δ*pde1*::spec Δ*bgaA*::P_{fucose}-*pde1*[T594I]-*tet*	Tet, Spec	This study
Sp_4383	D39 Δ*cps* Δ*pde1*::spec Δ*bgaA*::P_{fucose}-*pde1*[D418A]-*tet*	Tet, Spec	This study
Sp_4434	D39 Δ*cps* Δ*pde1*::spec Δ*bgaA*::P_{fucose}-*pde1*[H345Y]-*tet*	Tet, Spec	This study
Sp_0280	D39 Δ*cps* CEP::*kan*	Kan	This study
E. coli			
**Strain Name**	**Genotype/Sequence**	**Resistance Marker(s)**	**Source or Reference**
DH5α *λpir* '0002'	*fhuA2*, *Δ(argF-lacZ)U169*, *phoA glnV44*, *Φ80, Δ(lacZ)M15, gyrA96, recA1, relA1, endA1, thi-1, supE44, deoR, hsdR17, λpir* phage lysogen.	-	Gibco BRL
4036	DH5α λ*pir* pCMK20501	Tet, Amp	This study
4067	DH5α λ*pir* pCMK20505	Tet, Amp	This study
4069	DH5α λ*pir* pCMK20506	Tet, Amp	This study
4071	DH5α λ*pir* pCMK20507	Tet, Amp	This study
4073	DH5α λ*pir* pCMK20508	Tet, Amp	This study
4075	DH5α λ*pir* pCMK20509	Tet, Amp	This study
4099	DH5α λ*pir* pCMK20510	Tet, Amp	This study
**Plasmids**			
**Plasmid Name**	**Genotype/Sequence**	**Resistance Marker(s)**	**Source or Reference**
pAKF205	*bgaA*::P_{fucose}- (*tet) bla pACYC ori*	Tet, Amp	(3)
pCMK20501	*bgaA*::P_{fucose}-*pde1* (*tet) bla pACYC ori*	Tet, Amp	This study
pCMK20505	*bgaA*::P_{fucose}-*pde1*[D418A] (*tet) bla pACYC ori*	Tet, Amp	This study
pCMK20506	*bgaA*::P_{fucose}-*pde1*[A78S] (*tet) bla pACYC ori*	Tet, Amp	This study
pCMK20507	*bgaA*::P_{fucose}-*pde1*[Q339H] (*tet) bla pACYC ori*	Tet, Amp	This study
pCMK20508	*bgaA*::P_{fucose}-*pde1*[C549R] (*tet) bla pACYC ori*	Tet, Amp	This study
pCMK20509	*bgaA*::P_{fucose}-*pde1*[T594I] (*tet) bla pACYC ori*	Tet, Amp	This study
pCMK20510	*bgaA*::P_{fucose}-*pde1*[H345Y] (*tet) bla pACYC ori*	Tet, Amp	This study
**Oligonucleotides**			
**Name**	**Genotype/Sequence**		
AntibioticMarker_F	GAGGGAGGAAAGGCAGGA		
AntibioticMarker_R	CGCCGTATCTGTGCTCTC		
*bgaA*_PznDiagnostic_F	GTTGCTACTAAACGTCTTCAAGGACG		
*pde1*_3FLANK_F	**GAGAGCACAGATACGGCG**GTACAACGGATTATGGAAGAGTTAGGC		
*pde1*_3FLANK_R	CTCGAATGTTCTTAAACCCGCTTCG		
*pde1*_5FLANK_F	TTGGAAGAAGCCGGTGCTAAGG		
*pde1*_5FLANK_R	**TCCTGCCTTTCCTCCCTC**GGATAAGACTCCAAACGCAATCAATCC		
*pde1*_A78S_F	**GAAGCGTTATTCTGTTCATATG**GATGCTTCTTCCGGTGTTTTG		

*pde1*_A78S_R	**CATATGAACAGAATAACGCTTC**TCACCAAGCTTGGCATAAGTAGACGG
Pde1_C549R _R3	**TAATTTACGCCCCTG**TAAAATAAGTTCATTAACCTCACGATATTCTTCAAAATCTGTCGCAGCAATTTC
Pde1_C549R_F3	**CAGGGGCGTAAATTAGG**TTCAGATGTACTAATAGCAGAGGCTAAGGACACGAAATGC
*pde1*_D418A_F	**GATTCTTGTAGCCCATTCAAAG**ACAGCCTTAACATTATCAAAAG
*pde1*_D418A_R	**CTTTGAATGGGCTACAAGAATC**AACAAAGAACGATTGGTCACC
*pde1*_H345Y_F	**TGTAGTCGGTTACAAAAATT**TAGACATGGATGCTTTGGGC
*pde1*_H345Y_R	**AATTTTTGTAACCGACTA**CAAAAACCTGATCTACACTCCG
*pde1*_nativeProm_F_3	TATACC<u>CTCGAG</u>GTGTAAAGGTAGGTTTACTG
*pde1*_nativeRBS_F	TAA<u>CTCGAG</u>CCAAGAGGTTTTGAATGAAAAAATTTTATGTAAGTCC
*pde1*_ORF_BamHI_R	**GCTTCGGCGGGGTTTTTTTTT<u>GGATCC</u>TT**TCATTCTTCTTTCTCCTTTTCC
*pde1*_ORF_R	ATA<u>GGATCC</u>CATCATTCTTCTTTCTCCTTTTCCTTTATTTC
*pde1*_Q339H_F2	**GGTATGCACTTGTTCGCC**AGCAATGTGATTGAAAATAGCTATGCTCTTTATGATGAAG
*pde1*_Q339H_R2	**GGCGAACAAGTGCATACC**TACAGCAGAGCCCAAAGCATCCATGTCTAAATTTTTG
*pde1*_seq_F	GCGCTTGGGGACAGAACTTCC
*pde1*_seqF_700 bp	CTGACTACACGGTGCTTGAGG
*pde1*_T594I _R2	AATCCTTGTATATTCTTCGCAAGAACAAAACTCGCTTCAATACCTGACATGG
*pde1*_T594I_F2	**GCGAAGAATATACAAGGATTT**ATCTCTATCTCAGCTCGAAGTCGTAGTAAACTG
*Pde1*_XhoI_optRBS_F	**GATTATAATAAAGTTAATCAAGGACG<u>CTCGAG</u>**GGGTGCCA**G**GAGGTTTTGGAATGAA
*pde2*_3FLANK_F	**GAGAGCACAGATACGGCG**GCAAGTGGTGCTAATTCCTATAGCC
*pde2*_3FLANK_R	GGCTAAAAGCTGGTTACTAGTTTTAGC
*pde2*_5FLANK_F	GGAAAGTCTGGACTGGCTGGTAACC
*pde2*_5FLANK_R	**TCCTGCCTTTCCTCCCTC**GCATCAGGGTCTGGTTTCATATGACG
*pde2*_seq_F	GCTAAAATCAAGCCCTTACTAGTCCCC
Pfuc_BamHI_F	ATA<u>GGATCC</u>AAAAAAAAACCCCGCCGAAGC
Pfuc_XhoI_R	ATA<u>CTCGAG</u>CGTCCTTGATTAACTTTATTATAATC
pLEM019_seq_F	CTAAATATTTCACAGCGACATTCACG
pLEM019_seq_R	GTTGTCATACCATGTATACCACTTGG
pLEM023_F	CACGATTACTTGGGGATCTCCCCGCGAAAGCGGG
pLEM023_R	TCCTGCCTTTCCTCCCTCGTCATACCATGTATACCACTTGG
*rplI*_3FLANK_F	**GAGAGCACAGATACGGCG**GGTTGGTTTGATTGATGTGCCAGTG
*rplI*_3FLANK_R	CGAATCTCTGTAATCCGAATACCTGGC
*rplI*_5FLANK_F	CAGATAAGATTCGGAGTGTAGATCAGG
*rplI*_5FLANK_R	**TCCTGCCTTTCCTCCCTC**CGCATACCCTGTTGGTACTTCC
*rplI*_seq_F	GACCAGGTGGTTGTTAAGGAAAACG
*rpmE2*_3FLANK_F	**GAGAGCACAGATACGGCG**GGACGTCAAAAGTTCACTCAAGCAG
*rpmE2*_3FLANK_R	CCTTTGGTCGTTACAAGGAAAGCGG
*rpmE2*_5FLANK_F	CGTCATATGAAACCAGACCCTGATGCC
*rpmE2*_5FLANK_R	**TCCTGCCTTTCCTCCCTC**CCATGAAGACAACTGGGCGATATTC
*rpmE2*_seq_F	GGTATTACCGGTCATACTGG
CEP_3FLANK_F	**GAGAGCACAGATACGGCG**CTACACAAAAATAGGCTCCATAATATCTATAAGGG
CEP_3FLANK_R	GGAATTGACTCGATAGCTTTAACATTAGCC
CEP_5FLANK_F	CTCAGGCGGTCAACGTCAACG
CEP_5FLANK_R	**TCCTGCCTTTCCTCCCTC**CGAAATTTGTCCTTTCTCGAGCTTAGC

CEP_SEQ_F	GCTGAGCACTTGACTCGTTACC

The D39 Δ*cps* genotype (Δ*cps2A'*-Δ*cps2H'*) was excluded from derivative strains for clarity. Strains are ordered as introduced in the manuscript, whilst plasmids and oligonucleotide sequences are shown in alphabetical order. Amp = ampicillin, Cam = chloramphenicol, Erm = erythromycin, Kan = kanamycin, Spec = spectinomycin, Tet = tetracycline, CEP = 'chromosomal expression platform'. *S.pn* = ORF amplified from *S. pneumoniae* D39. For oligonucleotide sequences; where restriction enzyme sites have been introduced these are underlined. Any primer containing relevant sequence from an ORF are shown in blue. Overlapping sequences used for isothermal assembly when generating gene knockout or genome integration constructs are shown in **bold**. Regions where codons have been altered are highlighted in red.

References

1. J. A. Lanie *et al.*, Genome Sequence of Avery's Virulent Serotype 2 Strain D39 of *Streptococcus pneumoniae* and Comparison with That of Unencapsulated Laboratory Strain R6. *J. Bacteriol.* **189**, 38-51 (2007).
2. A. Prjibelski, D. Antipov, D. Meleshko, A. Lapidus, A. Korobeynikov, Using SPAdes de novo assembler. *Current Protocols in Bioinformatics* **70**, e102 (2020).
3. K. A. Jolley, M. C. J. Maiden, BIGSdb: Scalable analysis of bacterial genome variation at the population level. *BMC Bioinformatics* **11**, 595 (2010).
4. K. Jolley, J. Bray, M. Maiden, Open-access bacterial population genomics: BIGSdb software, the PubMLST.org website and their applications [version 1; peer review: 2 approved]. *Wellcome Open Research* **3** (2018).
5. A. K. Fenton, L. El Mortaji, D. T. C. Lau, D. Z. Rudner, T. G. Bernhardt, CozE is a member of the MreCD complex that directs cell elongation in *Streptococcus pneumoniae*. *Nature Microbiol.* **2**, 16237 (2016).
6. R. R. Chaudhuri, M. J. Pallen, xBASE, a collection of online databases for bacterial comparative genomics. *Nucleic Acids Res.* **34**, D335-D337 (2006).
7. R. R. Chaudhuri *et al.*, x BASE2: a comprehensive resource for comparative bacterial genomics. *Nucleic Acids Res.* **36**, D543-D546 (2007).
8. D. G. Gibson *et al.*, Enzymatic assembly of DNA molecules up to several hundred kilobases. *Nat. Methods* **6**, 343-345 (2009).
9. M. J. Boersma *et al.*, Minimal Peptidoglycan (PG) Turnover in Wild-Type and PG Hydrolase and Cell Division Mutants of *Streptococcus pneumoniae* D39 Growing Planktonically and in Host-Relevant Biofilms. *J. Bacteriol.* **197**, 3472-3485 (2015).
10. E. Kuru *et al.*, In Situ probing of newly synthesized peptidoglycan in live bacteria with fluorescent D-amino acids. *Angew Chem Int Ed Engl* **51**, 12519-12523 (2012).
11. C. A. Schneider, W. S. Rasband, K. W. Eliceiri, NIH Image to ImageJ: 25 years of image analysis. *Nat. Methods* **9**, 671-675 (2012).
12. A. Ducret, E. M. Quardokus, Y. V. Brun, MicrobeJ, a tool for high throughput bacterial cell detection and quantitative analysis. *Nature Microbiol.* **1**, 16077 (2016).
13. S. S. Shapiro, M. B. Wilk, An analysis of variance test for normality (Complete Samples). *Biometrika* **52**, 591-611 (1965).
14. Student, The probable error of a mean. *Biometrika* **6**, 1-25 (1908).
15. Y. Bai *et al.*, Two DHH subfamily 1 proteins in *Streptococcus pneumoniae* possess cyclic di-AMP phosphodiesterase activity and affect bacterial growth and virulence. *J. Bacteriol.* **195**, 5123-5132 (2013).
16. S. Guiral *et al.*, Construction and evaluation of a chromosomal expression platform (CEP) for ectopic, maltose-driven gene expression in *Streptococcus pneumoniae*. *Microbiology (Reading)* **152**, 343-349 (2006).
17. F. Sievers *et al.*, Fast, scalable generation of high-quality protein multiple sequence alignments using Clustal Omega. *Mol. Syst. Biol.* **7**, 539-539 (2011).
18. S. R. Eddy, Profile hidden Markov models. *Bioinformatics* **14**, 755-763 (1998).

19. T. J. Wheeler, S. R. Eddy, nhmmer: DNA homology search with profile HMMs. *Bioinformatics* **29**, 2487-2489 (2013).
20. I. Letunic, P. Bork, Interactive tree of life (iTOL) v3: an online tool for the display and annotation of phylogenetic and other trees. *Nucleic Acids Res.* **44**, W242-245 (2016).
21. K. P. Schliep, phangorn: phylogenetic analysis in R. *Bioinformatics* **27**, 592-593 (2010).
22. D. L. Swofford, W. P. Maddison, Reconstructing ancestral character states under Wagner parsimony. *Math. Biosci.* **87**, 199-229 (1987).
23. J. Hoskins *et al.*, Genome of the bacterium *Streptococcus pneumoniae* strain R6. *J. Bacteriol.* **183**, 5709-5717 (2001).
24. T. J. Coffey *et al.*, Horizontal transfer of multiple penicillin-binding protein genes, and capsular biosynthetic genes, in natural populations of *Streptococcus pneumoniae*. *Mol. Microbiol.* **5**, 2255-2260 (1991).
25. L. K. McDougal *et al.*, Analysis of multiply antimicrobial-resistant isolates of *Streptococcus pneumoniae* from the United States. *Antimicrobial Agents and Chemotherapy* **36**, 2176-2184 (1992).
26. R. Muñoz *et al.*, Intercontinental spread of a multiresistant clone of serotype 23F *Streptococcus pneumoniae*. *J. Infect. Dis.* **164**, 302-306 (1991).
27. Z. Y. Shi, M. C. Enright, P. Wilkinson, D. Griffiths, B. G. Spratt, Identification of three major clones of multiply antibiotic-resistant *Streptococcus pneumoniae* in Taiwanese hospitals by multilocus sequence typing. *J. Clin. Microbiol.* **36**, 3514-3519 (1998).
28. G. Gherardi, C. G. Whitney, R. R. Facklam, B. Beall, Major related sets of antibiotic-resistant Pneumococci in the United States as determined by pulsed-field gel electrophoresis and *pbp1a-pbp2b-pbp2x-dhf* restriction profiles. *J. Infect. Dis.* **181**, 216-229 (2000).
29. S. S. Richter *et al.*, The molecular epidemiology of penicillin-resistant *Streptococcus pneumoniae* in the United States, 1994-2000. *Clin. Infect. Dis.* **34**, 330-339 (2002).
30. N. Porat, R. Trefler, R. Dagan, Persistence of two invasive *Streptococcus pneumoniae* clones of serotypes 1 and 5 in comparison to that of multiple clones of serotypes 6B and 23F among children in southern Israel. *J. Clin. Microbiol.* **39**, 1827-1832 (2001).
31. A. B. Brueggemann, B. G. Spratt, Geographic distribution and clonal diversity of *Streptococcus pneumoniae* serotype 1 isolates. *J. Clin. Microbiol.* **41**, 4966-4970 (2003).
32. M. C. Enright, B. G. Spratt, A multilocus sequence typing scheme for *Streptococcus pneumoniae*: identification of clones associated with serious invasive disease. *Microbiology (Reading)* **144 (Pt 11)**, 3049-3060 (1998).
